# Active and durable R_2_MnRuO_7_ pyrochlores with low Ru content for acidic oxygen evolution

**DOI:** 10.1038/s41467-023-37665-9

**Published:** 2023-04-10

**Authors:** Dmitry Galyamin, Jorge Torrero, Isabel Rodríguez, Manuel J. Kolb, Pilar Ferrer, Laura Pascual, Mohamed Abdel Salam, Diego Gianolio, Verónica Celorrio, Mohamed Mokhtar, Daniel Garcia Sanchez, Aldo Saul Gago, Kaspar Andreas Friedrich, Miguel A. Peña, José Antonio Alonso, Federico Calle-Vallejo, María Retuerto, Sergio Rojas

**Affiliations:** 1 Grupo de Energía y Química Sostenibles, Instituto de Catálisis y Petroleoquímica, CSIC. C/Marie Curie 2, 28049 Madrid, Spain; 2 Institute of Engineering Thermodynamics/Electrochemical Energy Technology, German Aerospace Center (DLR), Pfaffenwaldring 38-40, 70569 Stuttgart, Germany; 3 Departament de Ciència de Materials i Química Fisica & Institut de Química Teòrica i Computacional (IQTCUB), Universitat de Barcelona, Martí i Franqués 1, 08028 Barcelona, Spain; 4 Diamond Light Source, Harwell Science and Innovation Campus, Didcot, OX11 0DE UK; 5 Instituto de Catálisis y Petroleoquímica, CSIC. C/Marie Curie 2, 28049 Madrid, Spain; 6 Chemistry Department, Faculty of Science, King Abdulaziz University, P.O. Box 80200, Jeddah, 21589 Saudi Arabia; 7 Instituto de Ciencia de Materiales de Madrid, CSIC. C/Sor Juana Inés de la Cruz 3, 28049 Madrid, Spain; 8 Nano-Bio Spectroscopy Group and European Theoretical Spectroscopy Facility (ETSF), Department of Advanced Materials and Polymers: Physics, Chemistry and Technology, University of the Basque Country UPV/EHU, Avenida Tolosa 72, 20018 San Sebastián, Spain; 9 IKERBASQUE, Basque Foundation for Science, Plaza de Euskadi 5, 48009 Bilbao, Spain

**Keywords:** Electrocatalysis, Fuel cells, Electrocatalysis

## Abstract

The production of green hydrogen in water electrolyzers is limited by the oxygen evolution reaction (OER). State-of-the-art electrocatalysts are based on Ir. Ru electrocatalysts are a suitable alternative provided their performance is improved. Here we show that low-Ru-content pyrochlores (R_2_MnRuO_7_, R = Y, Tb and Dy) display high activity and durability for the OER in acidic media. Y_2_MnRuO_7_ is the most stable catalyst, displaying 1.5 V at 10 mA cm^−2^ for 40 h, or 5000 cycles up to 1.7 V. Computational and experimental results show that the high performance is owed to Ru sites embedded in RuMnO_x_ surface layers. A water electrolyser with Y_2_MnRuO_7_ (with only 0.2 mg_Ru_ cm^−2^) reaches 1 A cm^−2^ at 1.75 V, remaining stable at 200 mA cm^−2^ for more than 24 h. These results encourage further investigation on Ru catalysts in which a partial replacement of Ru by inexpensive cations can enhance the OER performance.

## Abstract (web summary)

Ru-pyrochlores find their way as alternative anodes of PEM water electrolyzers, and their high performance is owing to Ru sites embedded in RuMnO_x_ surface layers. Here, a water electrolyser with Y_2_MnRuO_7_ and only 0.2 mgRu cm^−2^ has been tested with significant durability.

## Introduction

Hydrogen produced from carbon-decoupled renewable sources, often referred to as “green” hydrogen, is expected to play a pivotal role in the transition to a carbon-neutral society. It allows to accumulate large amounts of renewable energy and can be applied in several hard-to-decarbonize sectors, including transport, industry (e.g., the production of ammonia and methanol or for that of stainless steel), and residential and industrial heat. Arguably, water electrolysis is one of the most efficient processes to cope with the storage of large amounts of intermittently-produced renewable electricity (in the order of hundreds of TWh) in the shape of hydrogen^1^. Electrolyzers are devices that generate H_2_ and O_2_ from H_2_O using electricity. In particular, proton exchange membrane water electrolyzers (PEMWE) are the most suitable ones for the production of green hydrogen from renewable energy^2^. In a PEMWE H_2_ is produced at the cathode, via the hydrogen evolution reaction (HER). However, because of the sluggish kinetics of the oxygen evolution reaction (OER), O_2_ production at the anode limits the process and is the main source of energetic inefficiencies in PEMWEs.

Because of the strongly oxidizing environment at which the OER takes place, i.e., low pH, high potential, and high O_2_ concentration, the state-of-the-art catalysts are based on iridium oxides, with Ir loadings typically in the range of 2.0–2.5 mg cm^−2^ ^3^. Nevertheless, Ir is one of the rarest elements on Earth, such that the anode is not only the greatest source of inefficiencies but also a major cause of the high costs of electrolyzers. In addition, the scarcity and market volatility of Ir can jeopardize the massive deployment of electrolyzers needed to cope with the projected production of green hydrogen, which is estimated to grow to some 350 GW by 2030^4^. Thus, it is imperative to design advanced electrocatalysts with reduced or no content of Ir at all. For instance, catalysts have been designed based on iridium-mixed oxides where a fraction of Ir is replaced by other metals. Perovskites, pyrochlores, spinels, and Ruddlesden-Popper phases with improved Ir mass-normalized OER activity have been reported^5–9^. The full replacement of Ir by other metals is more challenging. Ru nanoparticles and Ru_1-y_M_y_O_x_-based compounds^10–12^; and mixed Ru oxides, such as SrRuO_3_^13^, CaCu_3_Ru_4_O_12_^14^, or Cr_0.6_Ru_0.4_O_2_^15^, display high OER activities, but lack sufficient stability and durability. Furthermore, the partial replacement of Sr by monovalent alkaline metals such as Na or K has proven to increase the durability of perovskite oxides^16,17^.

Recently, Ir and Ru pyrochlores with general formula R_2_B_2_O_7_, where R is a rare-earth element, Y, Bi, or Pb; and B is mainly Ir and/or Ru; have been studied for the OER. For instance, Ir-pyrochlores such as Bi_2_Ir_2_O_7_, Pb_2_Ir_2_O_6.5_^18^, Pr_2_Ir_2_O_7_^8^, Y_2_Ir_2_O_7_^19^, display good OER activities. Mixed Ir/Ru pyrochlores have also been studied^9,20,21^. Several Ru pyrochlores have been evaluated, with different cations in the R position (R = Nd, Gd, Y, Bi, Sm, Er, Yb) and partial substitutions over that position, R_2-x_(Ba, Sr, *TM*)_x_Ru_2_O_7_ (*TM* = transition metals)^22–29^. However, Ru is also a scarce and expensive noble metal, such that attempts to reduce its content without incurring performance penalties, i.e., increasing Ru-mass-specific activity, ought to be investigated. To the best of our knowledge, there is one OER catalyst in which Ru is partially replaced by non-noble metal cations in the pyrochlore structure, Y_2_[Ru_1.6_Y_0.4_]O_7-δ_, showing higher activity than the parent Y_2_Ru_2_O_7-δ_ but without stability studies and a detailed understanding of the origin of its performance^30^.

In this work, we synthesized and studied the OER performance of R_2_MnRuO_7_ with R = Y, Tb, and Dy, a family of low-Ru-content pyrochlores where Ru is partially replaced by Mn cations in the B sites. R_2_MnRuO_7_ pyrochlores display remarkable OER activity and durability in RDE and MEA configurations, especially in terms of Ru-mass-normalized activities. Computational modeling concluded that the high OER activity stems from Ru sites at a layer of RuMnO_x_ formed at the surface of the pyrochlores upon dissolution of R cations.

## Results and discussion

### Crystallographic structure of the pyrochlores

**Fig. 1:**
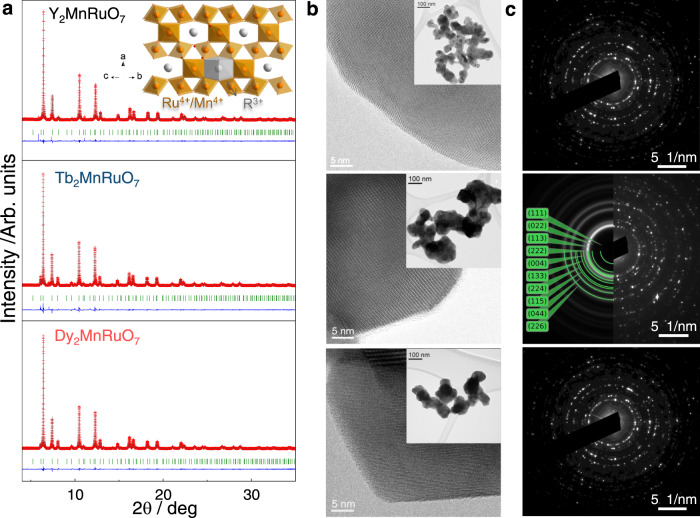
Morphological and structural characterization. **a** SXRD Rietveld analysis at room temperature of the pyrochlore structures. Inset: schematic view of the crystal structure of the pyrochlores. **b** HRTEM images. Insets: TEM images. **c** Electron diffraction of the pyrochlores under study.

The structure of the pyrochlores was analyzed by conducting Rietveld refinements of the SXRD data, see Fig. 1a. R_2_MnRuO_7_ oxides (R = Y, Tb, Dy) present a pyrochlore-type structure with formula A_2_B_2_O_7_ (cubic F*d-3m* space group). The active Mn and Ru sites are located at octahedral MnO_6_ and RuO_6_ positions sharing corners (inset Fig. 1a) which are not ordered between them. Tables S1 and S2 in the supporting information (SI) summarize the unit cell, atomic positions, occupancies, and main interatomic distances and angles for the three pyrochlores determined from SXRD at room temperature.

The morphology, particle size, and composition of the catalysts were studied by TEM. As shown in the inset of Fig. 1b, the samples are aggregates of small spheroid-like particles of ~60 nm (Figure S1). The HRTEM (Fig. 1b) shows crystalline planes in the pyrochlore and the absence of amorphous phases at the surface of the catalysts. The SAED images display diffraction spots corresponding to crystalline pyrochlores (Fig. 1c). The compositions obtained by EDX are in good agreement with the nominal ones, and similar to the occupancies determined from SXRD (Table S1).

### OER activity in RDE

**Fig. 2:**
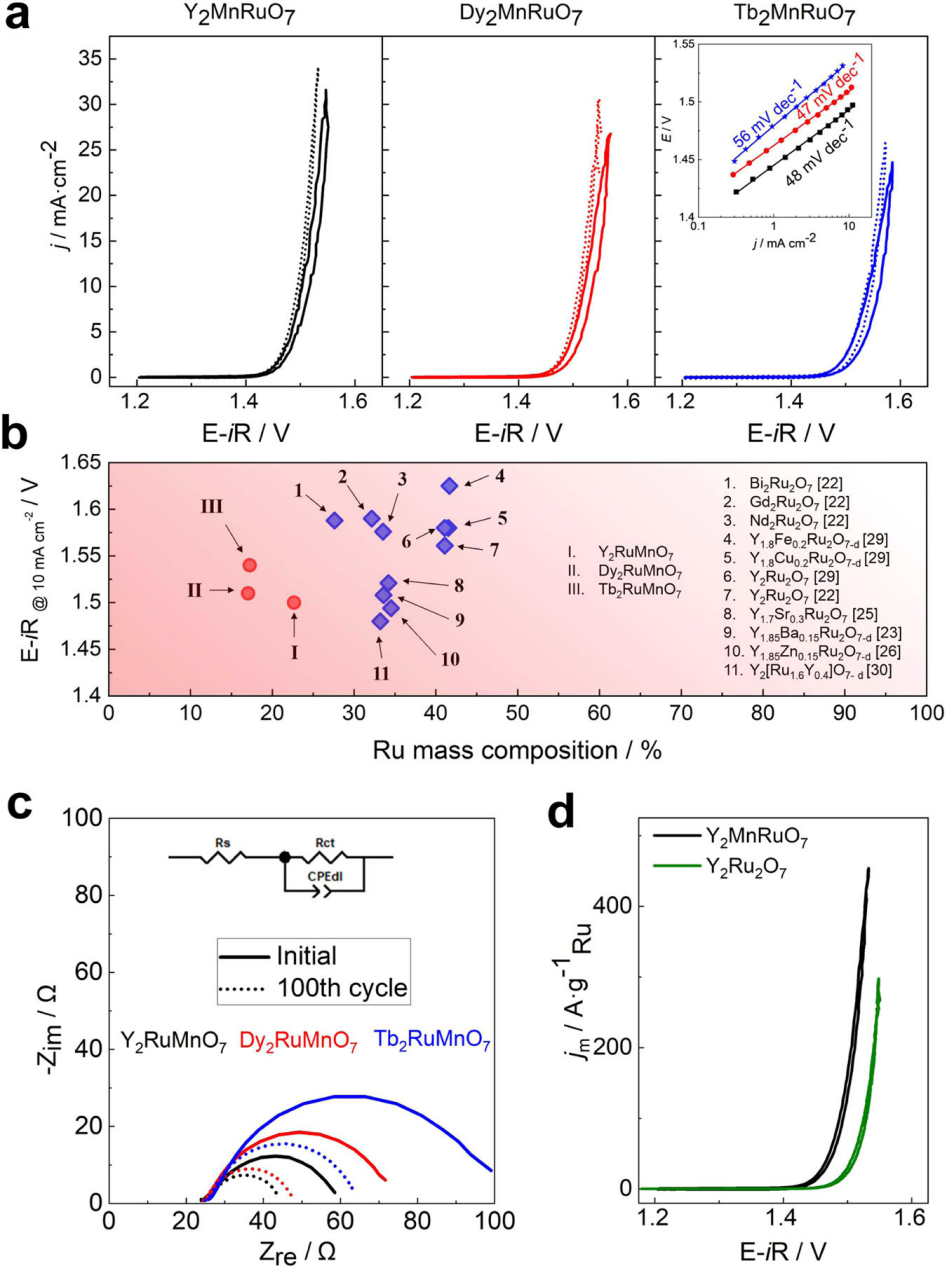
OER performance. **a** First (solid lines) and 100th (dotted lines) OER polarization curves for each catalyst recorded in O_2_-saturated 0.1 M HClO_4_ at 10 mV s^−1^ and 1600 rpm. Inset: Tafel plots after 100 cycles. **b** Performance of various Ru pyrochlores for the OER in acid electrolyte: those in this work are in red, and those in the literature are in blue (data source in brackets). **c** Comparative Nyquist plots recorded at 1.52 V vs RHE and proposed equivalent circuit. **d** The Ru-mass-specific activity of Y_2_MnRuO_7_ (black) compared to homemade Y_2_Ru_2_O_7_ (green).

Figure 2a shows the 1st and 100th OER polarization curves. A clear hysteresis between the anodic and cathodic sweeps is observed in the first voltammogram. The hysteresis decreases upon cycling and the OER activity increases slightly, becoming stable after 100 cycles. Most likely, the hysteresis stems from a side reaction leading to the surface reconstruction of the catalysts during the first cycles (see the Evolution of the catalysts during the OER section). This kind of restructuration has been reported for other Ru and Ir-pyrochlores^22^. In view of this, the 100th voltammogram is taken as the representative one to benchmark the activity of each catalyst. Potentials of 1.50, 1.51, and 1.54 V are needed to achieve a current density of 10 mA cm^−2^ for Y_2_MnRuO_7,_ Dy_2_MnRuO_7_, and Tb_2_MnRuO_7_, respectively. The inset of Fig. 2a depicts the Tafel plots for each catalyst after 100 cycles. Y_2_MnRuO_7_ and Dy_2_MnRuO_7_ display Tafel slopes of ~47 mV dec^−1^, whereas Tb_2_MnRuO_7_ records a Tafel slope of 56 mV dec^−1^. These values are similar to those reported for other Ru pyrochlores (see Table 1), suggesting that the OER proceeds by a similar mechanism in all catalysts. We note that Table 1 contains the highest OER activity for each catalyst in the cited manuscripts, regardless of the cycle at which such activity was recorded. The results in Fig. 2b reveal that the family of R_2_MnRuO_7_ is among the best Ru-mixed pyrochlore catalysts in acid electrolytes for the OER in the literature and their Ru content is appreciably lower.

**Table 1:** Performance of Ru pyrochlores for the OER in acidic electrolytes

	*j*/mA cm^−2^_geo_ @ 1.5 V	E-*i*R/V @ 10 mA cm^−2^_geo_	Tafel slope/mV dec^−1^	Ru mass-specific activity/A g^−1^_Ru_	Durability tests RDE
Y_2_RuMnO_7_ (this work)	12^†^	1.49^†^	48^†^	795 @ 1.55 V^†^	45 h @ E_j = 10_
Dy_2_RuMnO_7_ (this work)	6.5^†^	1.51^†^	47^†^	707 @ 1.55 V^†^	25 h @ E_j = 10_
Tb_2_RuMnO_7_ (this work)	2.5^†^	1.54^†^	56^†^	335 @ 1.55 V^†^	15 h @ E_j = 10_
Y_2_Ru_2_O_7_ (this work)	3.1^†^	1.52^†^	46^†^	245 @ 1.55 V^†^	
Y_2_Ru_2_O_7_^22^	4.7@1.55 V^‡*^	1.561(5)^‡^	40^‡^	~500 @ 1.55 V^*^	~ 8 h @ 1.56 V
Nd_2_Ru_2_O_7_^22^	2.6@1.55 V^‡*^	1.576(9)^‡^	41^‡^	~350 @ 1.55 V^*^	~ 4 h @ 1.56 V
Gd_2_Ru_2_O_7_^22^	2@1.55 V^‡*^	1.590(7)^‡^	47^‡^	~250 @ 1.55 V^*^	~ 6 h @ 1.56 V
Bi_2_Ru_2_O_7_^22^	1.9@1.55 V^‡*^	1.588(7)^‡^	48^‡^	~250 @ 1.55 V^*^	~6 h @ 1.56 V
Y_1.85_Ba_0.15_Ru_2_O_7-δ_^23^	6.9	1.508	40.8	-	1500 cycles bt. 1.4–1.6 V
Y_1.7_Sr_0.3_Ru_2_O_7_^25^		1.494	44.8	1018 @ 1.53 V	
Y_1.85_Zn_0.15_Ru_2_O_7-δ_^26^	4.4	1.521	36.9	-	2000 cycles bt. 1.35–1.6 V
Y_2_[Ru_1.6_Y_0.4_]O_7-δ_^30^	18.1	1.48	37	700 @ 1.5 V	-
Y_1.8_Fe_0.2_Ru_2_O_7-δ_^29^	0	1.625^*^	57–63	~400 @ 1.625 V^*^	-
Y_1.8_Cu_0.2_Ru_2_O_7-δ_^29^	>1	1.58^*^	57–63	~400 @ 1.55 V^*^	6 h @ 1 mA cm^−2^_geo_
Y_1.8_Ni_0.2_Ru_2_O_7-δ_^29^	>1	-	57–63	~400 @ 1.575 V^*^	-
Y_1.8_Co_0.2_Ru_2_O_7-δ_^29^	0	-	57–63	~400 @ 1.585 V^*^	-
Y_2_Ru_2_O_7-δ_^29^	>1	1.58^*^	57–63	~400 @ 1.55 V^*^	-
Yb_2_Ru_2_O_7_^9^				650 @ 1.58 V	-
RuO_2_^22^	1.2 @ 1.55 V	1.626 (14)	77		-

Unless otherwise stated, the activity of the first OER cycle is reported.

^*^Activity values extracted from the figures of the indicated references.

^‡^OER at 10th cycle.

^†^OER at the 100th cycle.

EIS analyses were carried out before and after 100 cycles at 1.52 V for each pyrochlore to further investigate the interfaces of the electrocatalysts (Fig. 2c). An equivalent circuit based on previous studies^31–33^ has been proposed, in which R_s_ is the electrolyte resistance and CPE_dl_ is the constant-phase element representing the double-layer capacitance. R_ct_ is the charge-transfer resistance and is related to the kinetics of the reaction. Lower R_ct_ values are indicative of easy electron transfer through the electrode-electrolyte interface^34^. In line with the evolution of the voltammograms shown above, R_ct_ decreases significantly after 100 cycles, suggesting that after the surface reconstruction the catalysts have more effective charge transfer, hence resulting in enhanced electrocatalysis. Besides, the evolution of the EIS follows the same trend as the OER activity, with lower resistance associated with more active catalysts: Y_2_MnRuO_7_ more active than Dy_2_MnRuO_7_, and Dy_2_MnRuO_7_ more active than Tb_2_MnRuO_7_ (Figure S2a).

The activities reached by the catalysts can be related to their crystallographic structure, including (Ru,Mn)–O distances, angles, oxygen vacancies, etc. For instance, Y_2_MnRuO_7_ has slightly shorter (Ru,Mn)–O bonds and slightly less bent Ru/Mn-O-Ru/Mn angles (see Table S2) than Dy_2_MnRuO_7_ and Tb_2_MnRuO_7_, which mirrors the OER activity trend. A recent study indicates that higher OER activities are attributed to shifts of the Ru 4d band center to lower values, a feature related to shortened Ru-O distances^14,35^. However, other reports claim that shorter Ru-O bonds lead to stronger interactions between Ru 4*d*-O 2*p* orbitals, resulting in lower activities (as for RuO_2_ or Bi_2_Ru_2_O_7_)^22^. The size of R cations and the lattice parameters have also been reported to affect the OER activity of pyrochlores, as well as the distortions of the structure, including distortions of RuO_6_ and MnO_6_ octahedra^8,9^. Moreover, Ru-O-Ru angles supposedly affect the activity as well as the conductivity. For instance, Ru–O–Ru angles greater than 133^o^ are necessary to facilitate metallic conductivity, which is important for the OER activity. Admittedly, none of our pyrochlores presents an angle higher than 133^o^, but the Y-based pyrochlore has larger Ru/Mn–O–Ru/Mn angles than the Dy-based pyrochlore. In turn, those angles in the Dy-based pyrochlore are larger than those of the Tb-based pyrochlore. Again, this trend coincides with the OER activity trend. Note that the OER activities of R_2_Ru_2_O_7_ pyrochlores with R = Y are higher than with other cations (see Fig. 2b).

The Ru content of the pyrochlores reported in this work is significantly smaller than that of similar pyrochlores in the literature^22–27^. Fig. 2d compares the Ru mass-specific activity of Y_2_MnRuO_7_ with that of Y_2_Ru_2_O_7_, using the same amount of catalyst. Y_2_MnRuO_7_ records 700 A g^−1^_Ru_ at 1.55 V. This Ru-mass activity is more than two times larger than that of Y_2_Ru_2_O_7_ (300 A g^−1^_Ru_ at 1.55 V) reported in this work (see Fig. 2d), the latter being similar to the one in the literature for Y_2_Ru_2_O_7_^22,27^. In addition, the Ru-mass activity of Y_2_MnRuO_7_ is also larger than the activity reported for Yb_2_Ru_2_O_7_ of 650 A g^−1^_Ru_ at 1.58 V^9^. The computational study presented later in this work shows that the presence of Mn on the surface of R_2_MnRuO_7_ enhances the OER activity of the Ru sites of the pyrochlores. The Ru mass-specific activities of the three studied catalysts are plotted in Figure S2b.

To evaluate the area-specific activity of the catalysts, the surface area of each catalyst needs to be assessed. Here we resorted to the electrochemical surface areas (ECSA) and the mass-specific surface areas (*A*_*S*_) calculated using TEM data and assuming that the particles are close to a spherical geometry^36,37^, see Table S3. Figure S2c, d shows that the area-specific activities follow the same activity trends observed before in which Y_2_MnRuO_7_ displays higher current densities throughout the entire polarization range.

### OER durability

**Fig. 3:**
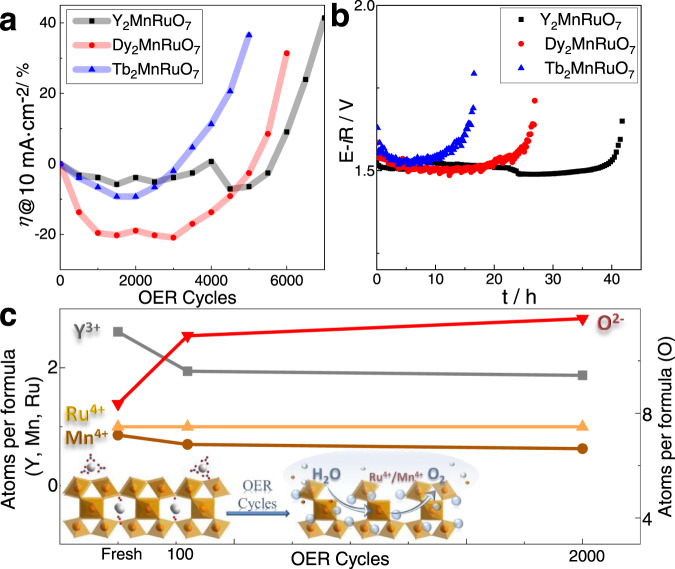
Durability of R_2_MnRuO_7_ pyrochlores under OER conditions. **a** Evolution of the potential at 10 mA cm^−2^ during OER cycles measured between 1.2 and 1.7 V at 50 mV s^−1^. **b** Chronopotentiometry measurements at a current density of 10 mA cm^−2^. **c** Surface composition of Y_2_MnRuO_7_ pyrochlore: initial catalyst, after 100 OER cycles and after 2000 OER cycles (data from Table S4). Lower inset: Schematic view of the surface reconstruction during the OER from XPS data. Orange circles and octahedra represent Ru^4+^/Mn^4+^ atoms and RuO_6_/MnO_6_, respectively, white circles represent Y^3+^, oxygen atoms are shown in red and C atoms from yttrium carbonate are shown in blue.

Catalyst durability was first assessed by recording CVs under OER conditions. As shown in Fig. 3a, the OER activity remains unaltered after the 100th cycle, remaining stable during 4500, 3500, or 2500 consecutive cycles for the Y-, Dy-, and Tb-containing pyrochlores, respectively. Chronopotentiometry durability tests were also performed (Fig. 3b). The current applied was set to 10 mA cm^−2^ and the evolution of the potential with time was monitored. The potential decreased during the first ca. 30 min of the experiment, in line with the hypothesized activation of the catalysts. Subsequently, the activity remained constant for ~15, 25, and 45 h for Tb_2_MnRuO_7_, Dy_2_MnRuO_7_, and Y_2_MnRuO_7_, respectively.

It is worth noting that the durability of these catalysts is remarkably high for Ru-based compounds. Most Ru oxides and perovskites with high OER activities deactivate after only a few OER cycles^13,16^. To the best of our knowledge, Ru pyrochlores are the only Ru-based catalysts endowed with high durability for the OER in acid. For instance, Jaramillo et al.^22^ reported a family of Ru pyrochlores with stable OER activity during 10 h at 10 mA cm^−2^. The Y_2_MnRuO_7_ pyrochlore reported in this work displays a stable OER activity during, at least, 40 h under the same experimental conditions (10 mA cm^−2^) despite having ca. half the content of Ru. This observation suggests that the presence of Mn at the surface of the catalysts results in both higher OER activity and durability.

### Evolution of the catalysts during the OER

The evolution of the pyrochlore structure and composition during the OER was analyzed both by in-situ and ex-situ characterization. First, we analyzed the evolution of the electrochemical surface area (ECSA) during cycling. The ECSA is directly related to the double-layer capacitance, C_dl_, and can be determined by CV or EIS. As shown in Figure S3, the double-layer capacitance increases during the first ca. 500 cycles, a feature associated with the reconstruction of the catalyst surface (see below). ECSA remains stable until ca. 5000 cycles, which is the region of stable OER activity, and finally drops with further cycling, coinciding with the catalyst deactivation profile.

The surface composition of Y_2_MnRuO_7_ and the oxidation states of Ru and Mn were studied by XPS. Spectra of the fresh catalyst and the catalysts recovered after 100 and 2000 OER cycles were collected. The surface atomic concentrations are proportional to the area under the curve of one of the characteristic peaks of each element (O 1 *s*, Y 3*d*, Ru 3*p*_3/2_, Mn 3*p*_3/2_) divided by the corresponding sensitivity factor^38^. We observe certain segregation of Y to the surface of the fresh catalyst. This feature has been reported for similar oxides with alkaline, alkaline-earth, and lanthanide cations, which tend to form surface carbonates in the presence of air^39^. The Y and O regions of the XPS corroborate the presence of carbonates (see Figure S4). The surface composition of the sample recovered after 100 OER cycles (Fig. 3c) indicates that the surface concentration of Y decreases, probably because the carbonate dissolves in the acid electrolyte but also because Y from the pyrochlore is partially dissolved. This observation agrees with the theoretical calculations (see below) where RuMnO_x_ layers are the active phase of the catalyst for the OER. After 2000 cycles the surface concentration is similar to that found after 100 cycles (Fig. 3c). The bulk elemental composition of Y, Mn, and Ru in Y_2_MnRuO_7_ after 2000 cycles has been also analyzed from EDX. Both 1 nm point EDX analyses and line profile analyses of the border of the particles reveal that the bulk stoichiometry of the pyrochlore is maintained in the particles. Note that it is difficult to determine any change of composition in the border of the particles beyond a slight Ru enrichment in some of them. The XPS regions of Ru and Mn (Figure S5) indicate that Ru^4+^ and Mn^4+^ are the main components of the pyrochlores and there are no significant variations in the oxidation state (or the shapes of the complexes) of these cations during the OER. Therefore, it seems that the final deactivation of the catalysts happens because the entire material degrades and dissolves, not due to the formation of different phases.

**Fig. 4:**
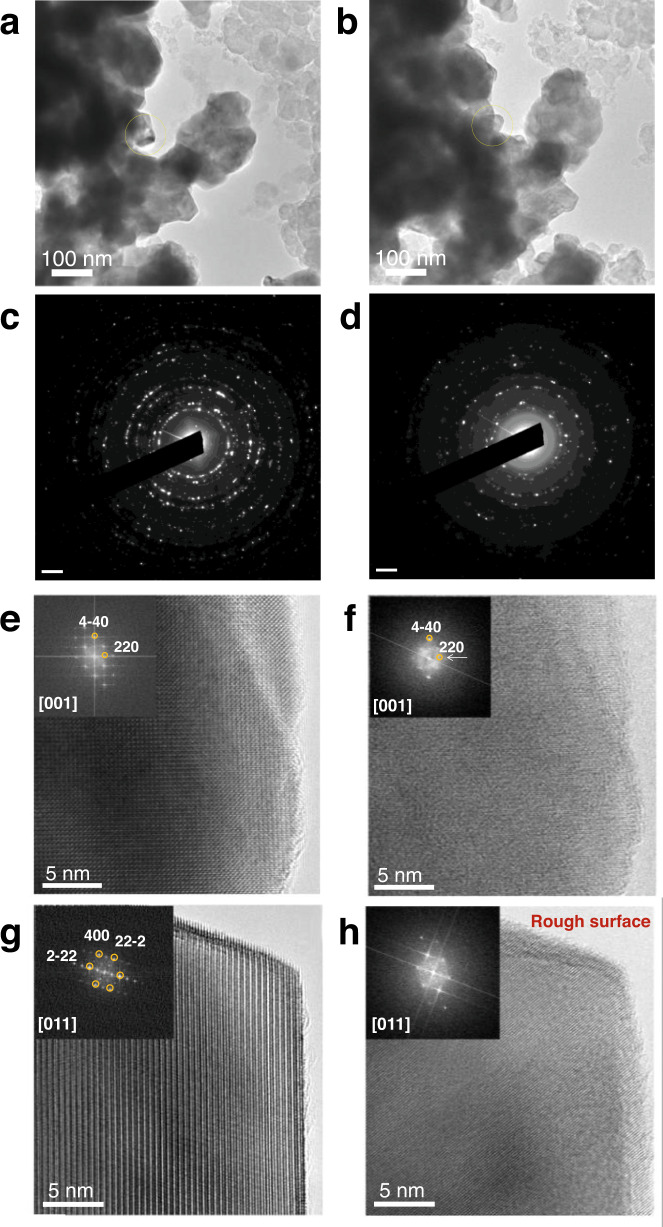
Identical locations TEM. Representative TEM images, **a** before and **b** after 2000 OER cycles. The corresponding SAEDS are shown in **c** before and **d** after 2000 OER cycles. HRTEM images of a particle oriented along the [011] zone axis **e** before and **f** after 2000 OER cycles. HRTEM images of a particle oriented along the [001] zone axis **g** before and **h** after 2000 OER cycles.

Representative IL-TEM images of Y_2_MnRuO_7_ before and after 2000 OER cycles are provided in Fig. 4. The morphology, shape, and size of the particles remain almost invariant after cycling. Subtle morphological changes are observed on some particles after 2000 cycles (see the yellow circles in Fig. 4a, b). The corresponding SAEDS (Fig. 4c, d) reveal only minor modifications at the microscale, as some diffraction spots disappear after the electrochemical reaction, but the crystallinity of the pyrochlore is preserved.

Further insights were obtained from the analysis of the HRTEM images of a particle oriented along the [011] zone axis (Fig. 4e, f). The surface of the cycled sample (panel f) is rougher than that of the fresh sample, indicating a higher level of disorder of the atoms located at the periphery of the particles. This disorder is manifested as a loss of crystallinity (symmetry), which is evident in the fast Fourier transform (FFT) images. After 2000 cycles (Fig. 4f, inset), the 111 and 200 reflections are no longer visible in some particles but the pyrochlore structure is still present. The loss of crystallinity seems to occur in all directions, as observed in other crystallographic directions (Fig. 4g, h), and is mainly restricted to the periphery of the particles.

**Fig. 5:**
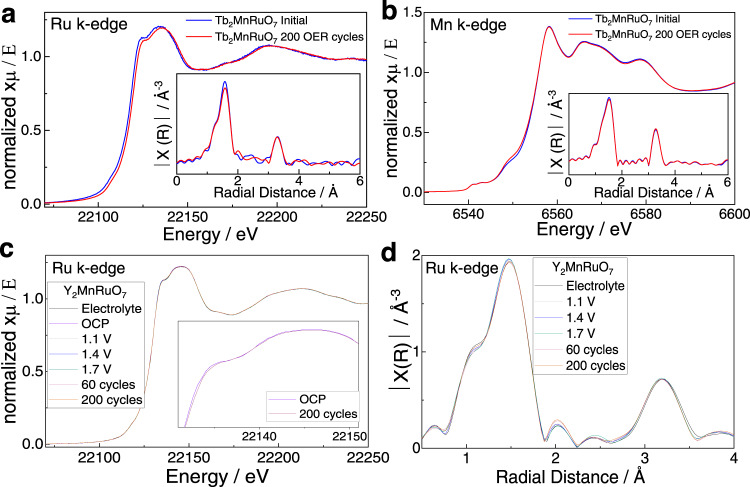
XAS measurements on Tb_2_MnRuO_7_ and in situ XAS on Y_2_MnRuO_7_. **a** Tb_2_MnRuO_7_ Ru K-edge XANES on fresh catalysts and catalysts after 200 OER cycles. Inset: EXAFS region. **b** Mn K-edge XANES spectrum for fresh Tb_2_MnRuO_7_ and after 200 cycles. Inset: EXAFS region. **c** Ru K-edge XANES spectrum for Y_2_MnRuO_7_ along cycling. Inset: zoom of the XANES of OCP and 200 cycles. **d** Ru K-edge EXAFS spectrum for Y_2_MnRuO_7_ along cycling.

XAS experiments were performed on Y_2_MnRuO_7_ and Tb_2_MnRuO_7_. Figure 5a, b shows the XANES and EXAFS Mn and Ru K-edge regions of Tb_2_MnRuO_7_ before and after 200 OER cycles. For Y_2_MnRuO_7_, we studied the evolution of the Ru K-edge during the OER in an in situ XAS experiment (Fig. 5c, d). The XANES spectra in both cases are almost invariant (Fig. 5a, c). Thus, no significant shifts due to changes in Ru oxidation state are observed. Only in the magnification of the XANES spectra of the Y-based pyrochlore at OCP and after 200 cycles (Fig. 5c, inset) is there a change of intensity of ~1%, which could indicate a slight geometric distortion in the coordination of Ru. This change can be explained by a different overlap of the orbitals, related to a slightly different configuration/environment of Ru at the surface of the particles. Such a change is only noticeable after 200 cycles, when the extent of surface reconstruction is more evident.

The EXAFS region spectra depict the typical distances of Ru and Mn cations in pyrochlores, which are maintained during the reaction. It is possible to observe the most intense 6-fold Ru-O coordination at 1.53 Å and both R-Ru and Ru-Ru coordination at 3.2 Å in both pyrochlores (Fig. 5a, d, insets). The presence of RuO_2_ would lead to two features at 2.6 Å and 3.2 Å, representing the twofold and eightfold Ru-Ru coordination. The absence of those features in the spectra indicates that RuO_2_ is not formed during the OER. It is important to note that since the particles are not in the nanometer range, XAS is not sensitive enough to accurately account for changes at their surface.

Finally, we determined the concentration of the cations in the electrolyte after 100 and 6000 OER cycles with Y_2_MnRuO_7_ (Table S5). After 100 cycles, we recorded a small dissolution of Y (8 wt.%) and Mn (4 wt.%). The amount of dissolved Ru is below the detection limit. After 6000 cycles, when the OER activity commences to decline, the dissolution of cations is more severe, and the fraction of dissolved metal accounts for 43, 18, and 9 wt.% for Y, Mn, and Ru, respectively. These results confirm the stability of the pyrochlore during the first 4000-5000 reaction cycles. They also confirm the faster dissolution of Y during the early reaction cycles, which is in line with the formation of Ru-Mn-O surface ensembles.

### Computational modeling of the OER

**Fig. 6:**
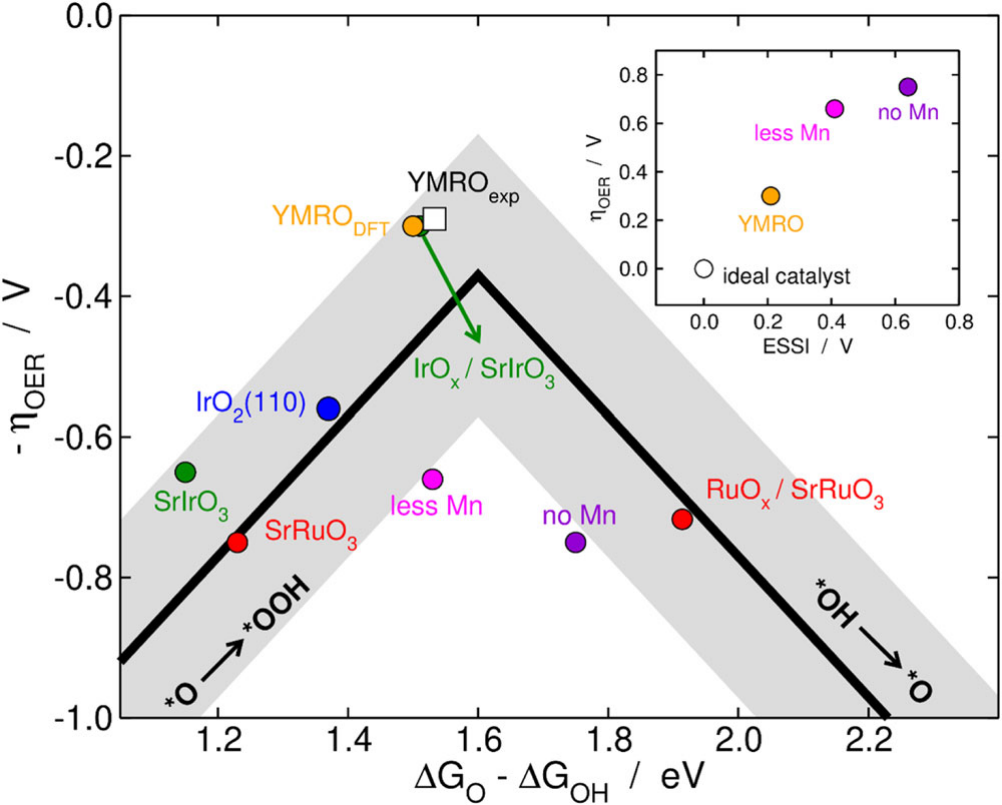
Volcano-type activity plot. OER activity in acid for a variety of oxides, including IrO_2_(110), pristine SrIrO_3_ and IrO_x_/SrIrO_3_^70^, pristine SrRuO_3_ and RuO_x_/SrRuO_3_^16^, and Y_2_MnRuO_7_ with a 1:1 ratio of Mn:Ru (denoted as YMRO), less Mn at the surface, and no Mn at the surface, see Figs. S7–S9. The white square (YMRO_exp_) is the experimental data from Fig. S2c adapted using a method reported elsewhere^16,66^, see section S1.2. Inset: OER overpotential as a function of the electrochemical-step symmetry index (ESSI) for different Ru-containing catalysts. The ideal catalyst, which has ΔG_i_ = 1.23 eV and *η*_OER_ = 0 V, is provided for comparison.

Seeking to elucidate the nature of the active sites and understand the effect of cycling on the OER activity of Y_2_MnRuO_7_, we carried out DFT calculations and semiempirical data analysis (see the Methods section and section S1.2). The specific values of the adsorption energies appear in Table S7. First, it is worth noting that the seminal work of Seitz et al.^40^ showed how the partial dissolution of non-noble elements in Ir-containing perovskites leads to an IrO_x_ skeleton with high OER activity (Fig. 6, green circles). However, for Ru-containing perovskites, the RuO_x_ skeleton is not more active than the pristine compound (Fig. 6, red circles). We do observe a high activity for Y_2_MnRuO_7_ upon partial dissolution of Y (Fig. 6, orange circle), which is in line with the experimental data from Figure S2c plugged into Fig. 6 (white square, the procedure is described in section S1.2). However, the progressive loss of Mn at the top layers of Y_2_MnRuO_7_ (magenta and purple circles in Fig. 6) increases the OER overpotential. In fact, the OER overpotential of the pyrochlore with no Mn at the top layers is close to that of RuO_x_/SrRuO_3_. This confirms that RuO_x_ skeletons are not remarkably active for the OER and suggests that the presence of Mn leads to a mixed RuMnO_x_ skeleton with high OER activity.

Moreover, the inset of Fig. 6 sheds light into the effect of Mn on the OER activity of Y_2_MnRuO_7_. The inset correlates the calculated OER overpotential with a metric for electrocatalytic symmetry called ESSI (electrochemical-step symmetry index)^41,42^, see section 2.8 and Table S7. As a reference, the ideal OER catalyst has *η*_OER_ = 0 V and ESSI = 0 V because all OER electrochemical steps take 1.23 eV (white circle), while real catalysts have ESSI ≥ 0.20 V. In addition, ESSI and η_OER_ are linearly related^41,42^. The inset of Fig. 6 shows that, initially, the 1:1 proportion between Ru and Mn ensures a high activity because the catalysts have well-balanced energetics of the electrochemical steps (orange circle), which corresponds to a low ESSI. As the content of Mn is lowered, the active sites become less energetically symmetric, which means that the magnitudes of the electrochemical steps are rather different and away from 1.23 eV and ESSI increases. In turn, alongside the increase of ESSI there is a concomitant increase of the OER overpotential (magenta and purple circles). In brief, we conclude that the OER active sites in our pyrochlores are made of Ru, but Mn is instrumental in their high activity.

In sum, our computational and experimental results indicate that Y_2_MnRuO_7_ pyrochlore is an excellent starting point to form an active RuMnO_x_ surface. Besides, we show here that a thoughtful design of the pyrochlore phases with suitable (Ru,Mn)–O bonds and Ru/Mn-O-Ru/Mn angles can provide active pyrochlores with lower Ru content than R_2_Ru_2_O_7_.

### Performance and stability of a Y_2_MnRuO_7_ anode for PEMWE

**Fig. 7:**
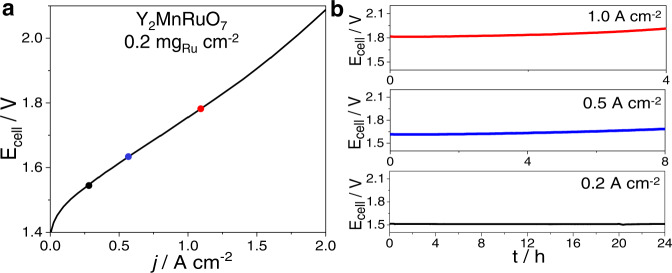
PEMWE measurements with Y_2_MnRuO_7_ anode (0.2 mg_Ru_ cm^−2^). **a** Cell potential (E_cell_) as a function of the current density (*j*) recorded galvanostatically up to 2 A cm^−2^ at 80 °C and 1 bar. **b** Durability tests of PEMWE at constant current densities of 0.2, 0.5, and 1 A cm^−2^ at 80 °C and 1 bar.

Since Y_2_MnRuO_7_ exhibits the highest OER activity and stability in RDE among the studied oxides, its OER performance was further assessed in a PEMWE electrolysis cell. Catalyst-coated membranes with Y_2_MnRuO_7_ as the anode catalyst and low Ru loadings of 0.2 mg_Ru_ cm^−2^ were produced by spray coating and subsequently tested in a PEMWE single cell with an active area of 4 cm^2^. Figure 7a shows the polarization curve recorded up to 2 A cm^−2^ at 80 °C and 1 bar. A PEMWE with a Y_2_MnRuO_7_ anode achieves 1.75 V at 1 A cm^−2^. The cell potential achieved lies within the state-of-the-art values for Ru-based catalysts^43–46^ and mixed Ir-Ru oxides^43,46–48^ tested in PEMWE (see also Figure S6 where a comparison with an Ir_black_ anode is performed). However, the scattered range of experimental conditions used in the literature prevents us from making a proper one-to-one performance comparison. Note that Ru-based catalysts, despite their good initial performance, are usually unstable under OER conditions, such that polarization curves at 1 A cm^−2^ or above are rarely reported. However, Y_2_MnRuO_7_ has been brought here up to 2 A cm^−2^ performing 2.08 V and showing a constant slope (see Fig. 7a). Also, it is worth emphasizing that most studies in the literature are carried out with significantly higher catalyst loadings, in the range of 1.5–3.0 mg cm^−2^. Our Y_2_MnRuO_7_ anode has about one order of magnitude lower precious metal loading, yet it reaches E_cell_ values comparable to other Ru-based electrodes.

Durability tests in single-cell configuration were conducted at different constant current values, see Fig. 7b. The cell maintains a constant potential of 1.51 V without losses at a current density of 0.2 A cm^−2^ for more than 24 h with a low degradation rate of 40 µA h^−1^. Further durability tests were carried out at higher constant current densities of 0.5 and 1 A cm^−2^. Under such high current densities, the potential finally goes up to 2 V. Nevertheless, this anode shows degradation rates of 7.5 mV h^−1^ and 22 mV h^−1^ during 4 h and 8 h, respectively. Although the durability of the PEMWE with an Y_2_MnRuO_7_ anode is lower than that reached with state-of-the-art Ir-based catalysts^49,50^, the durability displayed by our catalyst is higher than that reported for other Ru-based catalysts^10,45^, especially at high current densities (>200 mA cm^−2^), a feat achieved using a considerably lower Ru loading.

In perspective, this work shows that the activity and durability of Ru-based catalysts for the OER in acid are improved substantially by suitably mixing Mn and Ru within a pyrochlore structure. This is a step forward in the path toward the eventual development of stable and cost-effective Ru anodes for PEMWEs.

We studied catalysts R_2_MnRuO_7_ with R = Y, Tb, and Dy for the OER in acid media. These compounds contain ~50% less Ru than R_2_Ru_2_O_7_ while displaying superior OER performance. In fact, the Ru mass-specific OER activity in the acid electrolyte is as high as 700 A g^−1^_Ru_ at 1.55 V for Y_2_MnRuO_7_. Moreover, Y_2_MnRuO_7_ remains active for more than 5000 OER cycles up to 1.7 V and during 40 h of chronoamperometry test at 10 mA cm^−2^.

The characterization of the catalysts shows the enhanced stability of the pyrochlore structure during OER. Our computational study determined that the active catalyst phase is a RuMnO_x_ layer formed at the surface of the oxide upon Y dissolution, which is consistent with the partial dissolution of Y observed experimentally. Y_2_MnRuO_7_ was tested in a MEA configuration obtaining a polarization curve comparable to those of other Ru catalysts but with a significantly lower Ru loading of only 0.2 mg_Ru_ cm^−2^. The durability of the anode with Y_2_MnRuO_7_ catalyst for 24 h at 0.2 A cm^−2^ is illustrated. Despite the low-Ru loading, it was shown to reach current densities as high as 0.5 and 1 A cm^−2^ without abrupt drops in performance. This brings about a significant improvement at the substantially low Ru loading of 0.2 mg_Ru_ cm^−2^.

In brief, we showed here that the performance of Ru for the OER in acid can be substantially improved when embedded with Mn and a rare-earth element in a pyrochlore structure, especially in Y_2_MnRuO_7_. This finding opens a route to further enhance the activity and durability of Ru mixed oxides by partially replacing Ru by other cations, as potential electrocatalysts for the anode of PEMWEs with lower content of noble metals.

## Methods

### Synthesis of R_2_MnRuO_7_ pyrochlores

R_2_MnRuO_7_ oxides (M = Y, Tb, Dy) were prepared by the citrate method. Stoichiometric amounts of MnCO_3_, RuO_2_, and the R precursor (Y_2_O_3_, Tb_4_O_7_ or Dy_2_O_3_) were dissolved in 100 mL of citric acid and 5 mL of concentrated nitric acid. The solution obtained was slowly dehydrated at 120 °C, leading to the formation of an organic resin containing a homogeneous distribution of the cations. The resins obtained were dried at 180 °C, the organic materials were decomposed and the nitrates eliminated in a treatment at 600 °C for 12 h and, subsequently, at 800 °C for 2 h in air. The resulting materials were finally heated at 900 °C for 12 h under 200 bar of oxygen pressure to obtain the pure pyrochlore oxides without the competitive RMnO_3_ oxides (containing Mn^3+^).

### X-ray powder diffraction (XRD) and synchrotron X-ray powder diffraction (SXRD)

XRD was obtained in a Bruker-axs D8 Advanced diffractometer (40 kV, 30 mA), controlled by a DIFFRACT^PLUS^ software, in Bragg-Brentano reflection geometry with Cu K_α_ radiation (λ = 1.5418 Å). The data were obtained between 2θ values of 10 and 64° in steps of 0.05°. SXRD patterns were collected on the powder diffraction station of the MSPD beamline at the ALBA synchrotron, Barcelona (Spain), with 38 keV energy, λ = 0.3252 Å, and with the high angular resolution MAD set-up. The SXRD data were analyzed by the Rietveld method using the Fullprof program^51,52^. A pseudo-Voigt function was used to generate the line shape of the diffraction peaks. The following parameters were refined in the final run: scale factor, background coefficients, zero-point error, pseudo-Voigt corrected for asymmetry parameters, positional coordinates and isotropic thermal factors for all the atoms.

### Transmission electron microscopy (TEM)

For the TEM and IL-TEM (Identical Locations TEM) experiments (TEM, HRTEM, STEM-EDX analysis) a 200KV JEOL STEM/STEM 2100 F with an Oxford Instruments X-max80 EDX detector was used. For the TEM histograms approximately 200 micrographs of each sample were taken to ensure that the obtained data, morphology, and particle size are representative of each sample. The samples for IL-TEM were prepared by sonicating the ink and depositing a drop of it in a conventional lacey carbon Au-grid. The evolution of the exact same selected regions was studied under the TEM. The grid was placed in the RDE, after 200 cycles of electrochemical reaction, the TEM grid was rinsed with water and the same locations were found under the beam and studied under the same conditions.

### X-ray photoelectron spectroscopy (XPS)

XPS was collected with a VG Escalab200R electron spectrometer equipped with a Mg-Kα (hυ = 1253.6 eV) X-ray source. The catalysts were dispersed in a Nafion-free ink and deposited on a carbon double-side adhesive tape supported on a stainless-steel holder. The same holder-supported catalyst was used in different electrochemical treatments: fresh catalyst, 5 and 1000 OER cycles (between 1.2 and 1.7 V at 50 mV s^−1^). After every treatment, the catalyst was washed with water, dried at room temperature, and outgassed at 10^−6^ mbar in the XPS pre-chamber during 1 h. Then, the sample was transferred into the analysis chamber where a pressure of 10^−8^ mbar was reached. The C1*s* peak due to the carbon double-sided adhesive tape and the carbon Vulcan added to the ink was used as a reference (set at 284.6 eV). Peak intensities were estimated by calculating the integral of each peak after subtraction of a Shirley-shaped background and fitting the experimental peaks to a combination of Lorentzian and Gaussian curves.

### X-ray absorption spectroscopy (XAS)

XAS measurements were performed at room temperature at Diamond Light Source (UK) on the B18 beamline^53^. Data were collected at Mn K-edge (*E* = 6539 eV) and Ru K-edge (*E* = 22117 eV) using a double crystal Si311 monochromator and Pt-coated mirrors. The measurements were performed in transmission mode using as detector three ion chambers with a gas mixture of Ar or Kr and He (200 mbar Ar, 220 mbar Kr, 220 mbar Kr resulting in absorption of ca. 15%, 70%, 70%, respectively).

For the in situ XAS measurement, the sample was loaded into a custom-made electrochemical cell, available on the B18 beamline and with a design based on the cell developed by Wise et al.^54^. The electrode was prepared by drop-casting the catalyst ink (Y_2_MnRuO_7_/Vulcan) onto a carbon paper support (Toray Carbon Paper, PTFE treated, TGP-H-60, Alfa Aesar). Electrochemical measurements were collected with an IVIUM potentiostat, using a Pt wire as counter electrode and a Ag/AgCl reference electrode. Measurements were collected in 0.1 M HClO_4_ electrolyte. In situ XAS data were collected in the energy range from 21917 to 23117 eV with a continuous QEXAFS acquisition mode and a constant energy step of 0.5 eV. The duration of a single scan was ca. 3 min. The scans were repeated hundreds of times while cycling potentials were applied to the sample.

XAS data treatment (including normalization, extraction of c(k) and Fourier Transform) was performed with the Athena software from Demeter package^55^. For the analysis of trends on the whole series of data a custom Python script was used to monitor position and intensity of the normalized spectra white line.

### Inductively coupled plasma optical emission spectrometry (ICP-OES)

An ICP-OES PlasmaQuantÆ PQ 9000 Analytik Jena spectrometer was used for the analyses. Specimens for analysis were taken directly from the 0.1 M HClO_4_ electrolyte. The maximum possible concentration of cations was calculated from the loading of the catalyst and the volume of the electrolyte.

### Electrochemical characterization

An Autolab PGstat 302 N potentiostat/galvanostat was used to test the electrochemical performance of the oxides. The measurements were performed using a standard three-compartment glass cell and a rotating disk electrode (RDE) (Pine Research Instruments). A graphite bar and an Ag/AgCl (3 M) were used as counter and reference electrodes, respectively. The catalysts were deposited as inks on top of the working electrode. The composition of the ink was 5 mg_oxide_, 1 mg_vulcan_, 0.03 mL_Nafion_, and 0.97 mL_THF_. Vulcan is used to improve electrical conductivity. The solids were ultrasonically dispersed in tetrahydrofuran (THF) and Nafion (Nafion 117 ~5%, Sigma-Aldrich) using an Ultrasonic Processor UP50H (Hielscher). 10 μL of ink were dropped onto a glassy carbon electrode of 0.196 cm^2^ of area, with a catalyst loading of 0.25 mg_oxide_ cm^−2^.

To measure the oxygen evolution reaction, cyclic voltammograms were recorded between 1.18 and 1.7 V vs RHE at 10 mV s^−1^. Measurements were repeated at least three times using freshly deposited catalysts (including catalysts from different batches) to assess reproducibility. The measurements were performed in an O_2_ saturated 0.1 M HClO_4_ electrolyte to assure the O_2_/H_2_O equilibrium at 1.23 V, at a rotation rate of 1600 rpm. The OER polarization curves were *i*R-corrected by using the formula E-*i*R_corrected_ = E_applied_ – *i*R. In this formula, *i* is the current and R is the ohmic electrolyte resistance (R~25 Ω) as obtained from electrochemical impedance spectroscopy (EIS).

### Computational details

The DFT calculations were carried out using the VASP program suite^56^ with the projector augmented-wave (PAW) method^57^ and the RPBE exchange-correlation functional^58^. A cutoff energy of 400 eV for the plane-wave basis set was used and the geometries were relaxed until the forces remaining on the atoms were less than 0.05 eV Å^−1^. Due to complexity of the systems, the calculations were performed spin-restricted as a first approximation. We used the GGA + U approach, with U_eff_ = 6.70 eV on the Ru atoms, found by Kitchin and coworkers for Ru^4+^ by means of a linear-response method^59^; and U_eff_ = 5.00 eV on the Mn atoms, found by Ceder and coworkers for Mn^4+^ and Mn^3+^ through a self-consistent evaluation method^60^. The Methfessel-Paxton method^61^ was used to smear the Fermi level with k_B_*T* = 0.2 eV and the energies were extrapolated to 0 K. The k-points for the overlayer structures used as the model for the OER active surface were chosen as Monkhorst-Pack grids^62^ with a sampling of 4 × 4 × 1. A vacuum region of more than 16 Å was used to separate the periodic images of the slabs and dipole corrections were applied in the vertical direction. H_2_O and H_2_ were calculated in 15 × 15 × 15 Å^3^ boxes, sampling the Γ-point only and making use of Gaussian smearing and k_B_T = 0.001 eV.

The free energies of adsorption of the OER intermediates, namely, *O, *OH, and *OOH were approximated as: $\Delta {G}_{{ads}}\approx \Delta {E}_{{ads}}+\Delta {ZPE}-T\Delta S$, where $\Delta {E}_{{ads}}$ is the DFT-calculated binding energy, $\Delta {ZPE}$ is the change in zero-point energy calculated with DFT within the harmonic oscillator approximation, and $T\Delta S$ are the entropy corrections, which are only vibrational for adsorbates and include all sorts of contributions for H_2_ and H_2_O. The energetics of proton-electron pairs was approximated by means of the computational hydrogen electrode approach^63^. The aqueous environment of the electrochemical system was approximated using VASPsol^64,65^, with the standard settings for bulk water. The method used to build the partially dissolved structures is described in section S1.2 and the converged coordinates are given in section S1.3. The final model surface with *O, *OH, and *OOH is shown in Figs. S7–S8, and Fig. S9 shows the slabs with varying Mn content.

To be able to compare with previous works^16,40,66^, the OER pathway was assumed to be: H_2_O → *OH → *O → *OOH → O_2_, such that $\Delta {G}_{1}=\Delta {G}_{{OH}}$, $\Delta {G}_{2}=\Delta {G}_{O}-\Delta {G}_{{OH}}$, $\Delta {G}_{3}=\Delta {G}_{{OOH}}-\Delta {G}_{O}$ and $\Delta {G}_{4}=\Delta {G}_{{O}_{2}}-\Delta {G}_{{OOH}}$. In turn, $\Delta {G}_{{OH}}$, $\Delta {G}_{O}$ and $\Delta {G}_{{OOH}}$ are defined using water and protons and electrons as references. The OER overpotential was calculated as: ${\eta }_{{OER}}={{\max }}\left(\Delta {G}_{1},\Delta {G}_{2},\Delta {G}_{3},\Delta {G}_{4}\right)/{e}^{-}-1.23$. As shown in previous works^16^, experimental OER datapoints can be added to computational volcano plots using the semiempirical volcano of Seh et al.^66^. The ESSI is a metric for electrocatalytic symmetry and is calculated as^41,42^: ${\eta }_{{OER}}=\frac{1}{n}{\sum }_{i=1}^{n}(\Delta {G}_{i}^{+}/{e}^{-}-{U}^{0})$, where $\Delta {G}_{i}^{+}$ are the free energies of the electrochemical steps larger than 1.23 eV.

### MEA measurements using a Y_2_MnRuO_7_ anode

The catalyst-coated membranes (CCMs) were prepared by the wet spraying technique using a vacuum heating table (Fuel Cell Store) to hold the Nafion 212 PEM substrate in place and heat it to 100 °C during catalyst deposition. The distance between spraying nozzle and substrate was kept at. 6 cm, and the ink deposition rate was limited to 2–3 min mL^−1^. The inks were prepared by mixing 1 mg of catalyst in 1 mL of ultra-pure H_2_O (MiliQ, 18 MΩ cm^−1^) and the desired amount of Nafion® D521 solution (5 wt.% in lower aliphatic alcohols and water) to achieve an ionomer content of 25 and 30 wt.% for the anode and cathode layers, respectively. The mixture was sonicated for at least 1 h until the catalyst was well dispersed. 1 mL of isopropanol (IPA, ACS reagent, ≥99.5%) was added and the mixture was sonicated for 10 min to reach the adequate dispersion and homogeneity of the ink. This process was scaled up to the desired volume of ink. Subsequent to spraying and drying, the CCM was hot pressed at 5 Mpa and 125 °C. The result is a CCM with Y_2_MnRuO_7_ (0.2 mg_Ru_ cm^−2^) at the anode and Pt/C 40% (0.4 mg_Pt_ cm^−2^) at the cathode.

The CCMs were tested in a PEMWE setup optimized for screening cell components. On both the anode and cathode sides, a Ti porous sintered layer (PSL) on Ti mesh (PSL/mesh-PTL) compound PTL produced by diffusion bonding coated with Pt was deployed^67,68^. On the cathode, a carbon paper sheet (Spectracarb 2050A-1050) was used as an additional layer contacting the cathode catalyst layer on one side, and the PTL on the other side. On both the anode and cathode sides Ti-BPPs were employed. The cell-active area was 4 cm^2^ and tests were carried out at 80 °C and ambient pressure. The polarization curves were measured galvanostatically according to the JRC EU-harmonized procedure^69^, employing a dwell and consecutive recording period of 10 s for each current step. PEMWE polarization curves up to 2 A cm^−2^ and durability tests at current densities of 0.2, 0.5, and 1 A cm^−2^ were recorded.

## Supplementary information

Supplementary Information

Peer Review File

## Data Availability

The data that support the findings of this study are available within the article and its Supplementary Information files. All other relevant data supporting the findings of this study are available from the corresponding authors upon request.
