# Supplementary material for: Active and durable R2MnRuO7 pyrochlores with low Ru content for acidic oxygen evolution
Source: Nat Commun. 2023 Apr 10;14:2010. doi: 10.1038/s41467-023-37665-9 (PMC10086044; doi:10.1038/s41467-023-37665-9)
Supplement: Supplementary file 1 — Supplementary Information [file 41467_2023_37665_MOESM1_ESM.pdf]

Supplementary Information

Active and durable R_2MnRuO_7 pyrochlores with low Ru content for acidic oxygen evolution

Dmitry Galyamin[1], Jorge Torrero[2], Isabel Rodríguez[1], Manuel J. Kolb[3], Pilar Ferrer[4], Laura Pascual[5], Mohamed Abdel Salam[6], Diego Gianolio[4], Verónica Celorrio[4], Mohamed Mokhtar[6], Daniel Garcia Sanchez[2], Aldo Saul Gago[2], Kaspar Andreas Friedrich[2], Miguel A. Peña[1], José Antonio Alonso[7], Federico Calle-Vallejo[3,8,9], María Retuerto[1,*], Sergio Rojas[1,*]

[1]Grupo de Energía y Química Sostenibles, Instituto de Catálisis y Petroleoquímica, CSIC. C/Marie Curie 2, 28049, Madrid, Spain

[2]Institute of Engineering Thermodynamics/Electrochemical Energy Technology, German Aerospace Center (DLR), Pfaffenwaldring 38-40, 70569 Stuttgart, Germany

[3]Departament de Ciència de Materials i Química Fisica & Institut de Química Teòrica i Computacional (IQTCUB), Universitat de Barcelona, Martí i Franqués 1, 08028 Barcelona, Spain

[4]Diamond Light Source, Harwell Science and Innovation Campus, Didcot, OX11 0DE, UK.

[5]Instituto de Catálisis y Petroleoquímica, CSIC. C/Marie Curie 2, 28049, Madrid, Spain

[6]Chemistry Department, Faculty of Science, King Abdulaziz University, P.O. Box 80200, Jeddah, 21589, Saudi Arabia.

[7]Instituto de Ciencia de Materiales de Madrid, CSIC. C/ Sor Juana Inés de la Cruz 3, 28049 Madrid, Spain

[8]Nano-Bio Spectroscopy Group and European Theoretical Spectroscopy Facility (ETSF), Department of Polymers and Advanced Materials: Physics, Chemistry and Technology, University of the Basque Country UPV/EHU, Avenida Tolosa 72, 20018 San Sebastián, Spain.

[9]IKERBASQUE, Basque Foundation for Science, Plaza de Euskadi 5, 48009 Bilbao, Spain.

Corresponding author e-mail addresses: m.retuerto@csic.es; srojas@icp.csic.es

Table of contents

S1. Supplementary Notes

S1.1 Supplementary experimental details……….………………………..2

S1.2 Supplementary computational details……….………………………10

S1.3 Supplementary Converged coordinates…………..………….………...15

S2. Supplementary References……………………………..……………………18

S1. Supplementary Notes

S1.1 Supplementary experimental details.

S1.1.1 SXRD Rietveld refinement results

R_2MnRuO_7 oxides (R = Y, Tb, Dy) present a pyrochlore-type structure with formula $A_2B_2O_7$. The crystal structure was refined in the face-centred cubic F*d-3m* space group, with origin at (1/8, 1/8, 1/8). R cations are placed at 16*d* (½, ½, ½) sites, which are eightfold coordinated within distorted cubes containing six equally spaced oxygen anions (O1) and two oxygen anions (O2) at a slightly shorter distance. Mn and Ru cations are located randomly at 16*c* (0,0,0) sites, six-fold coordinated in trigonal antiprisms (distorted octahedra) with all the six oxygen anions (O1) at equal distances. The two nonequivalent oxygen atoms are located at p O1 at 48*f* (x, 1/8, 1/8) and O2 at 8*b* (3/8, 3/8, 3/8) sites. A small fraction of Mn cations is located at the 16*d* positions together with R atoms. The occupancy of O1 and O2 oxygen atoms was also refined, giving a slight deviation from the full stoichiometry for Y_2MnRuO_7 and Tb_2MnRuO_7. Powder neutron diffraction studies of similar pyrochlores reported the presence of oxygen vacancies[1]. Table S1 summarizes the unit cell, atomic positions, occupancies, thermal parameters and discrepancy factors for the pyrochlores, and Table S2 contains the main interatomic distances and angles at all temperatures.

Table S1. Unit-cell parameters, atomic positions, occupancies, thermal factors and reliability factors of R_2MnRuO_7 in the cubic *Fd-3m* (no. 227) space group.

	**Y_2MnRuO_7**	**Tb_2MnRuO_7**	**Dy_2MnRuO_7**
**a(Å)**	10.0337(2)	10.0872(2)	10.0657(1)
**V (Å^3)**	1010.16(3)	1026.39(4)	1019.83(3)
**R/Mn**			
f_{occ} R/Mn (%)	84.3/15.7(2)	92.8/7.2(2)	81.9/18.1(1)
B(Å^2)	0.63(2)	1.03(2)	0.726(6)
**Mn/Ru**			
f_{occ} Mn/Ru (%)	33.7/66.3(2)	42.9/57.1(2)	31.3/68.7(1)
B(Å^2)	0.18(2)	-0.3(2)	0.24(1)
**O1**			
x	0.3319(2)	0.3384(4)	0.3342(2)
B(Å^2)	0.6(1)	0.8(2)	1.8(2)
f_{occ}	1	1	1
**O2**			
B(Å^2)	0.6(1)	0.8(2)	1.8(2)
f_{occ}	0.93(2)	0.70(2)	1
**Rel. factors**			
χ^2	1.46	3.27	1.50
**R_p(%)**	7.92	9.77	7.11
**R_{wp}(%)**	10.8	13.4	8.72
**R_{exp}(%)**	8.90	7.42	7.12

Table S2. Selected atomic distances (Å) and angles (deg) for R_2MnRuO_7 at room temperature.

	**Y_2MnRuO_7**	**Tb_2MnRuO_7**	**Dy_2MnRuO_7**
		**Distance/Å**	
**(R,Mn)-O1(x6)**	2.4479(8)	2.421(2)	2.442(1)
**(R,Mn)-O2(x2)**	2.17237(2)	2.18394(3)	2.17928(2)
**(Mn,Ru)-O1(x6)**	1.9547(7)	1.994(3)	1.9696(9)
		**Angles/º**	
**(Mn,Ru)-O1-(Mn,Ru)**	130.30(3)	127.23(5)	129.22(4)
**O1-(Mn,Ru)-O1**	82.67(4)	80.69(17)	81.97(5)

S1.1.2 Particle size distribution from TEM data.

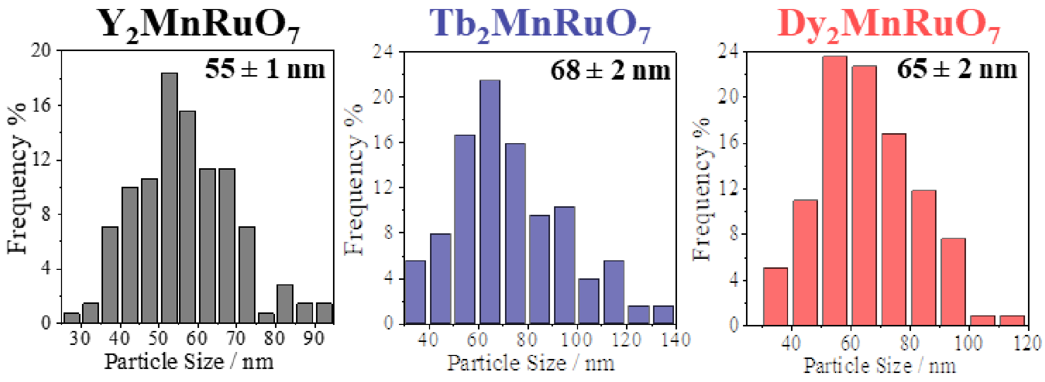

Figure S1. Histograms of particle-size distribution of the three pyrochlores under study.

S1.1.3 Additional electrochemical details

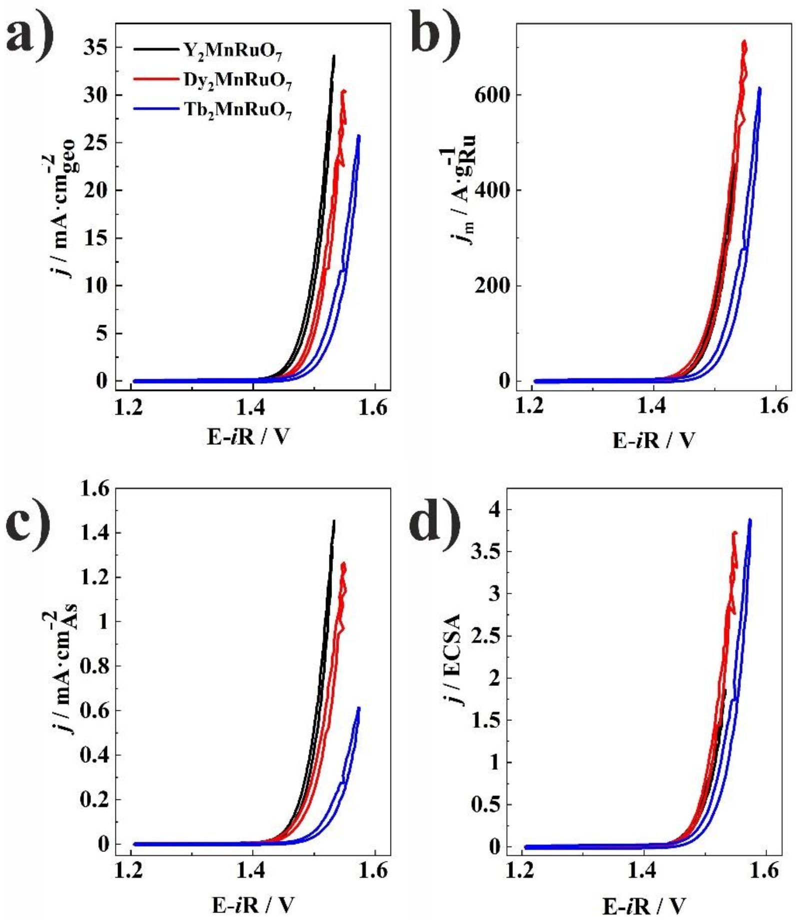

Figure S2. (a) Comparison of current densities at the 100th cycle between the pyrochlores. (b) Ru-mass specific activities of the pyrochlores (0.05 mg of catalyst on the electrode) The Ru-mass specific activity of (0.05 mg of catalyst on the electrode) the pyrochlores. (c) Activities normalized by A_s. (d) Activities normalized by ECSA.

S1.1.4 Electrochemical Active Surface Area

The electrochemical active surface area (ECSA) was based on the double-layer capacitance (C_{dl}) of the surface of the pyrochlores. Note that we prepare the regular inks but without adding vulcan. Cyclic voltammograms around the open circuit potential (OCP) in Ar were performed where the only processes occurring are supposed to be due to the double-layer charging. The cyclic voltammograms are carried out at 2, 5, 10, 25 and 50 mV s^{-1} and the double-layer charging current (i_c) is equal to the product of the scan rate (ν) and C_{dl} at a constant potential.

Plotting i_c *vs.* ν gives C_{dl} as the slope. Then ECSA is equal to C_{dl} divided by the specific capacitance (C_S) of an atomically flat planar surface of the compounds per unit area under the same electrolyte conditions. A typical value for C_S of 0.035 mF/cm^2 was used as it was previously used for several oxides in the same electrolyte.[2–4] The ECSA values are shown in Table S3.

S1.1.5 Assessment of the evolution of C_{dl} and ECSA with OER cycling

The experiments were conducted with Y_2MnRuO_7. An ink containing 0.05 $mg_{Y2MnRuO7}$, 0.01 mg_{vulcan}, 0.0003 mL_{Nafion} and 0.0097 mL_{THF} was dropped onto the working electrode to a final Ru loading of 0.011 mg. EIS experiments were recorded at open circuit potential after cycling.

ECSA is directly related to C_{dl}, and can be determined by CV or EIS. Both techniques provide similar information on the capacitive behaviour of the system. However, by measuring the capacitance behaviour by EIS, the C_{dl} can be replaced by a CPE and thus more information can be obtained, such as the evolution of the (non)-ideal capacitance of the surface. CPE can be expressed as: $Z_{CPE} = \frac{1}{(j\omega)^{\varphi} T_{dl}}$ (Eq. 1) where T_{dl} is a double-layer capacitance parameter, ω is the angular frequency of the ac perturbation and φ is the dimensionless CPE exponent representing the ideality or non-ideality of a capacitance. If φ = 1, the system acts as an ideal capacitance and as it decreases, so does the ideality.

The change on T_{dl} and φ with the OER cycle is given in Figure S3. Three main regions can be observed. In the first one, T_{dl} varies towards positive values due to the restructuring of the catalyst. In that same region, φ deviates to lower values indicating that the surface is less regular and, therefore, a less ideal capacity is implied. In the second region, the values of T_{dl} and φ remain constant. Finally, in the region of deactivation, the T_{dl} values drop sharply until the complete deactivation of the catalyst.

In the case of a redox reaction from the CPE, a value of C_{dl} can be obtained by the following equation:

$$C_{dl} = T_{dl}^{1/\varphi}\left(\frac{1}{R_s}+\frac{1}{R_{ct}}\right)^{1-1/\varphi} \qquad \text{(Eq. 2)}$$

The initial ECSA value was 9.3 cm^2, at the stable zone ECSA was around 12 cm^2 and the final value was 3.1 cm^2.

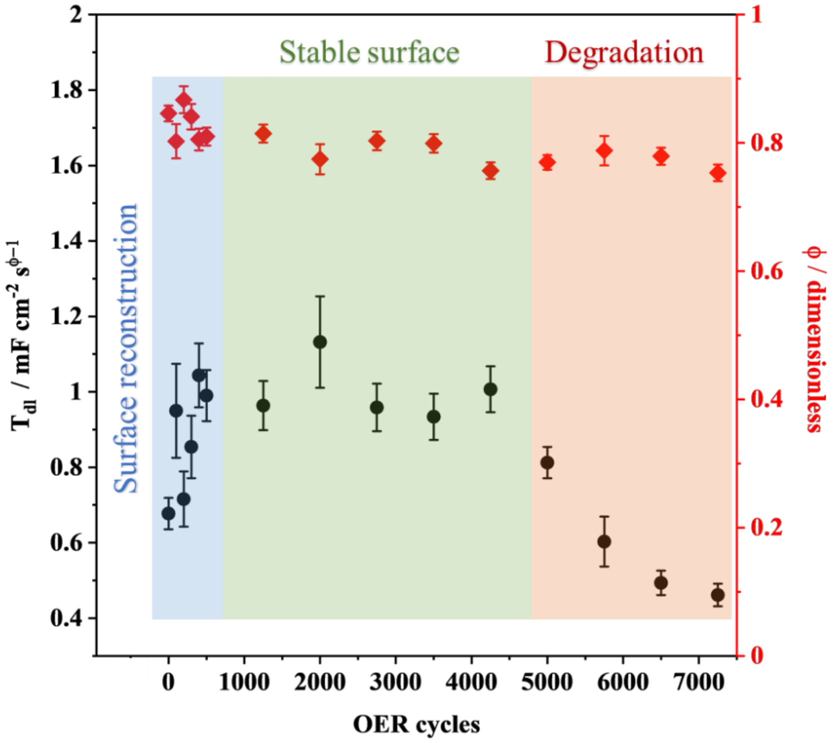

Figure S3. Evolution of the double-layer capacitance and φ with OER cycles. Three regions can be observed, surface reconstruction (blue), stable surface (green) and degradation (orange) in which the double-layer capacitance increases, remains stable and decreases, respectively.

S1.1.6 Mass-specific surface area (A_S) calculation

Mass-specific surface areas (A_S) are calculated using TEM data assuming that the particles are close to a spherical geometry.[5,6] A_S was determined using the following formula:

$$A_s = \sum \pi d^2 \Big/ \sum \tfrac{1}{6}\rho\pi d^3 = 6\sum d^2 \Big/ \rho \sum d^3 = 6/\rho d_{v/a} \qquad \text{(Eq. 3)}$$

where d are the diameters of each particle calculated by TEM, $d_{v/a}$ is the volume/area diameter ($d_{v/a} = \Sigma d^3/\Sigma d^2$), ρ is the pyrochlore bulk density ($\rho = Mw \cdot Z / N_A \cdot V_{f.u.}$, where Mw is the molecular weight, Z is the number of formula units per cell, N_A is Avogadro's number, and $V_{f.u.}$ is the volume of the formula unit).

Table S3. Surface area calculated by ECSA and As. TEM particle size. Amount of Ru in the electrode, both stoichiometric and calculated by EDX.

Property	Y_2MnRuO_7	Dy_2MnRuO_7	Tb_2MnRuO_7
**ECSA / cm^2**	3.6	1.6	1.3
**Gaussian TEM Particle size / nm**	55±2	65±2	68±2
**Mean TEM Particle size / nm**	56	66	73
**A_S / cm^2**	8.2	5.3	4.6
**mg_{Ru} in electrode (esteq.)**	0.0011	0.0085	0.0086
**mg_{Ru} in electrode (EDX)**	0.0015	0.0094	0.0083

S.1.1.7 X-Ray photoemission spectroscopy (XPS)

Table S4. Surface stoichiometry of Y_2MnRuO_7 using as a reference 1 Ru atom in the structure.

Surface stoichiometry	Y	Mn	O	Ru
**Fresh**	2.61	0.86	8.36	1.00
**100 OER cycles**	1.94	0.70	10.96	1.00
**2000 OER cycles**	1.87	0.63	11.61	1.00

Figure S4 shows the O 1*s* core-level region of the spectra of fresh Y_2MnRuO_7 and recovered after 100 and 2000 OER cycles. In the fresh catalyst the main contributor to the spectrum is the peak due to the pyrochlore lattice oxygen (O^{2-}). Secondary peaks appear mainly due to surface carbonates or hydroxides, most likely from Y carbonate, and adsorbed water.[7,8] After the OER tests, the components associated to hydroxides and adsorbed water clearly increase. The full width at half maximum (FWHM) of OH^-/CO_3^{2-} component also increases, indicating that a variety of surface hydroxides are formed that interact with different surface sites.

The Y 3*d* core-level spectrum of the fresh sample shows two features: the most intense at 156.6 eV can be ascribed to lattice Y^{3+}, while the minor component at 157.5 eV indicates the presence of Y carbonate in the fresh sample.[9,10] This component is slightly less intense after electrochemical treatment.

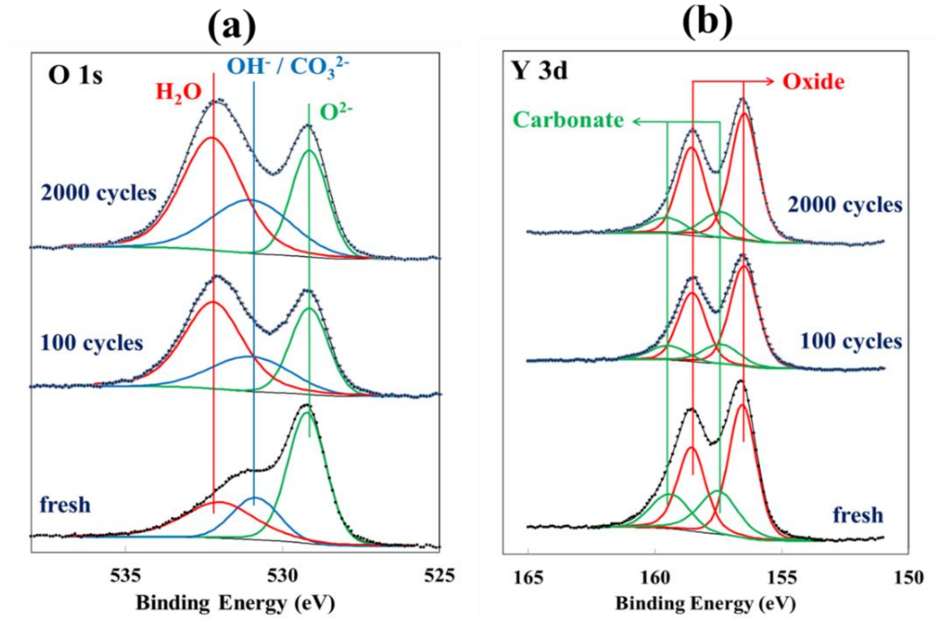

Figure S4. (a) O 1*s* core level, (b) Y 3*s* core level of Y_2MnRuO_7 pyrochlores: initial catalyst, after 100 OER cycles and after 2000 OER cycles.

Ru and Mn 3p lines are also doublet states (3$p_{1/2}$ and 3$p_{3/2}$), however, for this study only the 3$p_{3/2}$ are used (Figure S5). Ru^{4+} and Mn^{4+} are the main oxidation states in the fresh sample.[7,11–13] We did not observe a significant variation of the oxidation state of these cations during the OER, or a change in the shape or in the contributions observed for both cations. A shake-up satellite peak was considered in the fitting due to core-hole screening and/or the asymmetry of the main peak.[7,11] No significant variations in this satellite component were observed during the electrochemical characterization.

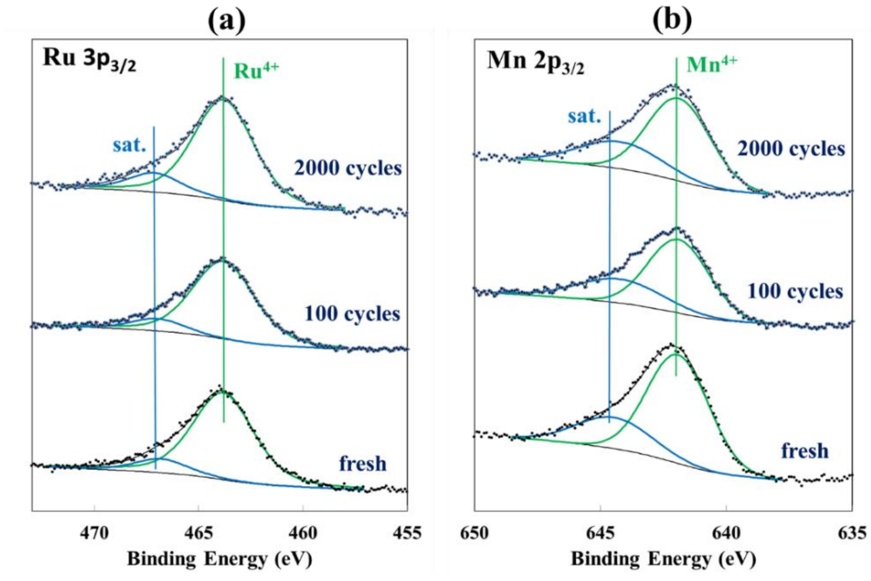

Figure S5. (a) Ru 3$p_{3/2}$ core level, (b) Mn 3$p_{3/2}$ core level of Y_2MnRuO_7 pyrochlores: initial catalyst, after 100 OER cycles and after 2000 OER cycles.

S.1.1.8 Inductively Coupled Plasma Optical Emission Spectrometry (ICP-OES)

The dissolution of cations from Y_2MnRuO_6 during OER cycling, at 100 and 6000 CV cycles, were determined by averaging two different experiments. The electrolytes were collected after both experiments and analysed by ICP-OES to determine the concentration of cations dissolved. Note that in the CV measurements we used 100 mL of electrolyte and 0.05 mg of pyrochlore. Therefore, the maximum concentration of cations that can be dissolved is 0.1994 mg L^{-1} of Y, 0.0661 mg L^{-1} of Mn and, 0.1134 mg L^{-1} of Ru.

Table S5. Concentration of dissolved cations obtained from ICP-OES measurements.

	**Element**	**Concentration / mg L^{-1}**	**Stantard Deviation**	**Cf***	**wt. % dissolved**
**Y_2MnRuO_7 100 cycles**	Y	0.0062	0.0003	0.0008	7.9
	Mn	0.0012	0.0006	0.0037	3.8
	Ru	0.0007	0.0007	0.0100	0.6
**Y_2MnRuO_7 6000 cycles**	Y	0.086	0.0007	0.0018	43.1
	Mn	0.012	0.0009	0.0037	18.2
	Ru	0.010	0.0007	0.0160	8.8

* Confidence limit with a confidence level of 95.4%.

S.1.1.9 Comparison of a PEMWE with an Y_2MnRuO_7 anode and a PEMWE with an Ir_{black} anode

The performance of a PEMWE cell with Y_2MnRuO_7 (0.2 mg_{Ru} cm^{-2}) was compared with that of a similar PEMWE with Ir_{black} (0.2 mg_{Ir} cm^{-2}). Stability tests at constant current densities of 0.2 and 0.5 A cm^{-2} were conducted. Figure S6 reveals that both PEMWE cells show similar performances at 0.2 A cm^{-2} for more than 24 h. At higher current densities, namely 0.5 A cm^{-2}, the PEMWE cell with Ir_{black} is stable, but the one with Y_2MnRuO_7 declines after 8 h, although it records lower potential values than the Ir_{black} catalyst for more than 8 h.

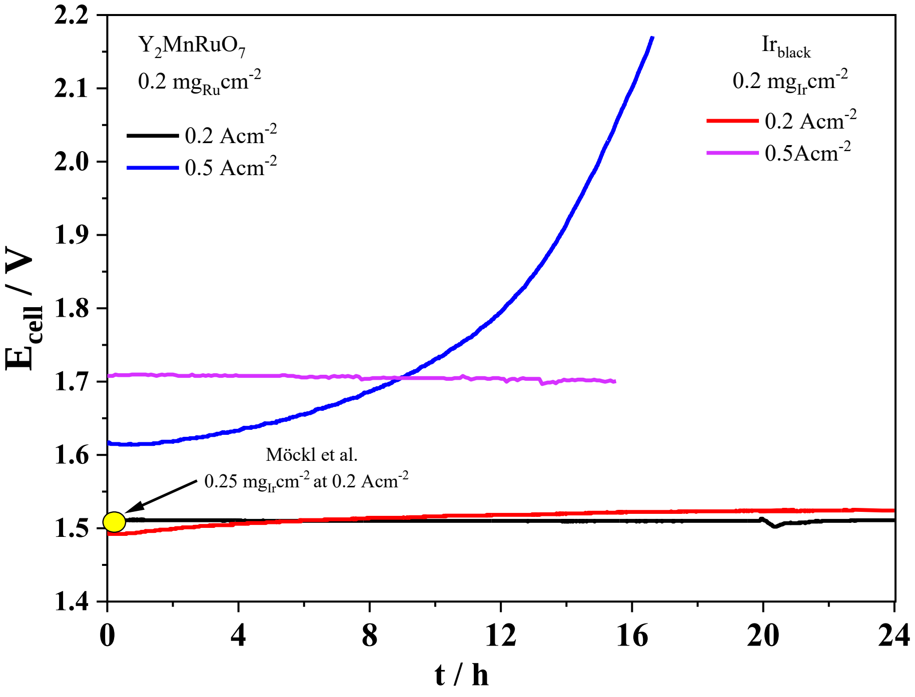

Figure S6. Stability test of PEMWE with Y_2MnRuO_7 or Ir_{black} anodes at constant current densities of 0.2 and 0.5 A cm^{-2} at 80 °C and 1 bar. The performance of a state-of-the-art MEA with 0.25 mg_{Ir} cm^{-2} by Möckl et al.[14] is shown for comparison.

For the sake of comparison, the activity of a state-of-the-art PEMWE cell with 0.25 mg_{Ir} cm^{-2} by Möckl et al.[14] is also shown. Although Möckl et al. demonstrate a long-term stability of the Ir catalyst, Y_2MnRuO_7 shows comparable performance during at least 24h, indicating an improvement over previous Ru-based anodes for PEMWE.

S1.2. Supplementary computational details

The surface structure of the dissolved Y_2MnRuO_7 pyrochlore was built consecutively by the following procedure:

I) We simulated the bulk structure of Y_2RuMnO_7.

II) We calculated the most stable surface facet of Y_2RuMnO_7 by calculating the surface energies for different surface terminations. We found that the (111) facet was the most stable, with a surface energy of 0.04 eV $Å^{-2}$, compared to the (100) facet with 0.06 eV $Å^{-2}$ and the (110) facet with 0.14 eV $Å^{-2}$.

III) We evaluated the OER performance of the sites on this surface and found onset potentials surpassing 2.00 V, which indicates that a pristine, stoichiometric slab is not representative of the surface present under experimental conditions.

IV) Since it is known that oxide surfaces with non-noble metals such as that of Y_2MnRuO_7 tend to leach metal atoms into the solution under acidic electrochemical conditions,[15,16] we then removed the Y atoms from the topmost surface layers.

V) From the structure described in the previous step, we calculated the adsorption energies of *O, *OH and *OOH on the Ru sites. The loss of Mn was calculated by replacing Mn by Ru in the topmost layers and re-relaxing.

Figure S7 shows top views of the converged geometries of the clean surface and the surface with *O, *OH and *OOH. Figure S8 shows the corresponding side views. Note that lattice oxygen atoms and oxygen atoms in the adsorbates are depicted in different colors. Figure S9 shows the varying amounts of Mn and Ru in the topmost layers of the slabs.

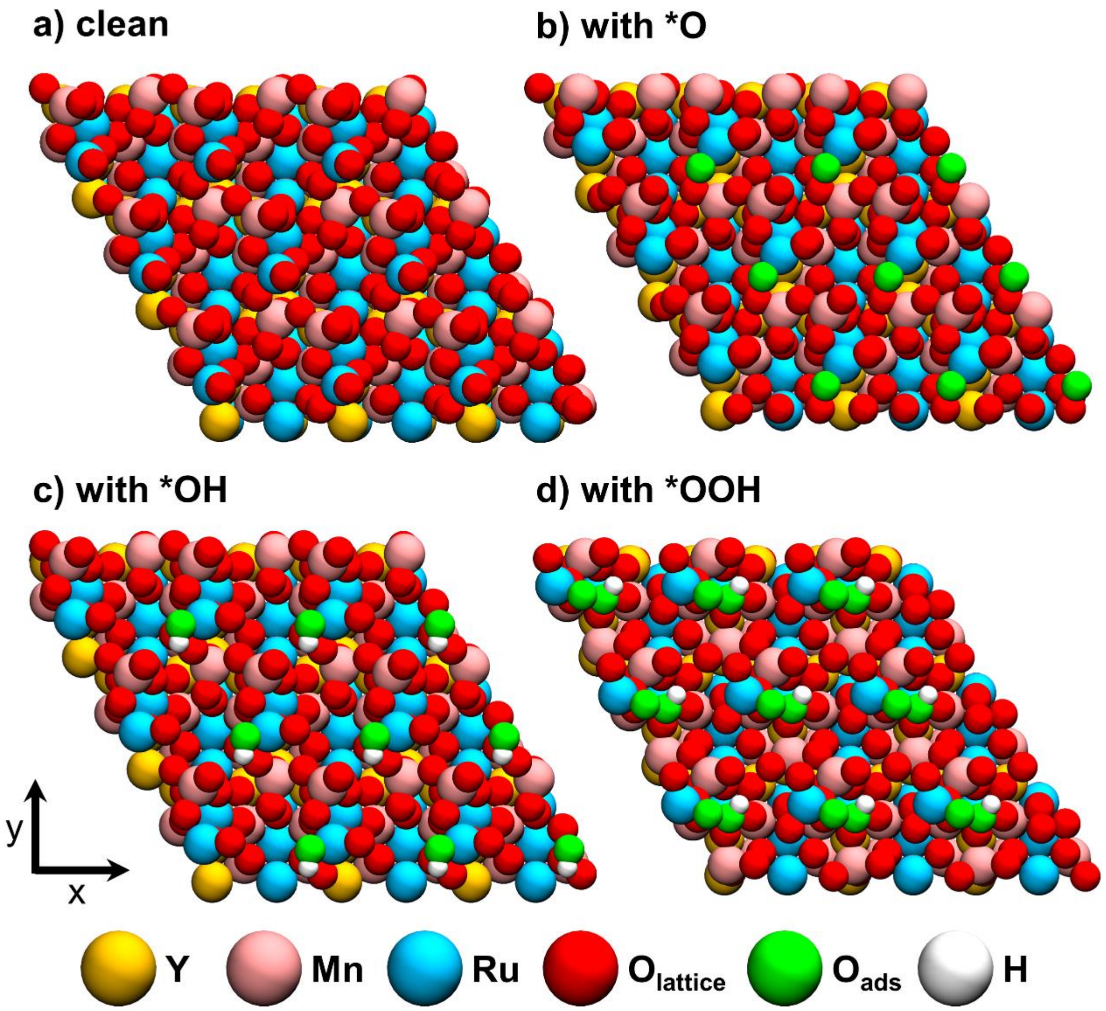

Figure S7. Top views of the relaxed slabs. The active site discussed in the main text is the topmost Ru site which the green O atoms are bound to. a) Clean slab. b) Slab with *O adsorbed on Ru. c) Slab with *OH adsorbed on Ru. d) Slab with *OOH adsorbed on Ru.

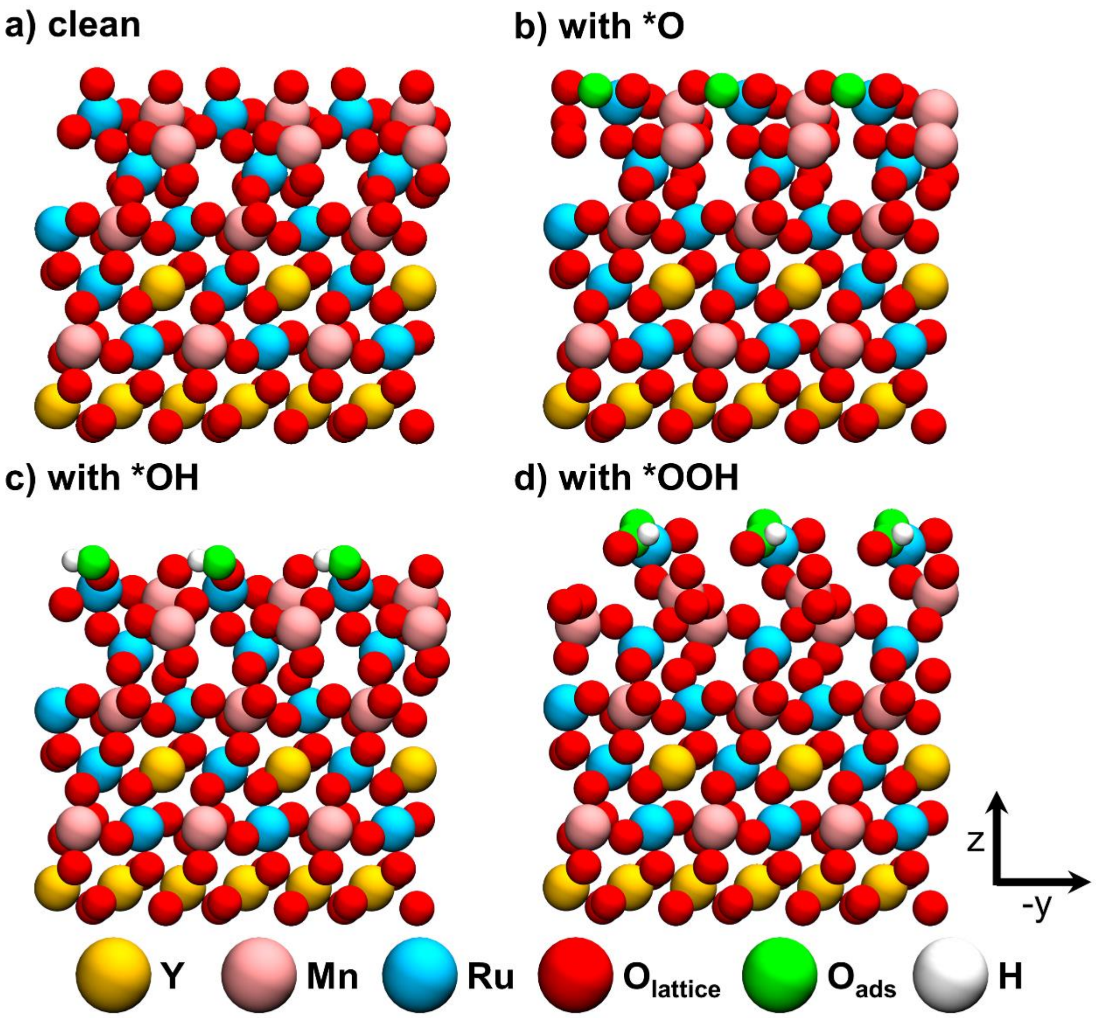

Figure S8. Side views of the relaxed slabs. The active site discussed in the main text is the topmost Ru site which the green O atoms are bound to. a) Clean slab. b) Slab with *O adsorbed on Ru. c) Slab with *OH adsorbed on Ru. d) Slab with *OOH adsorbed on Ru.

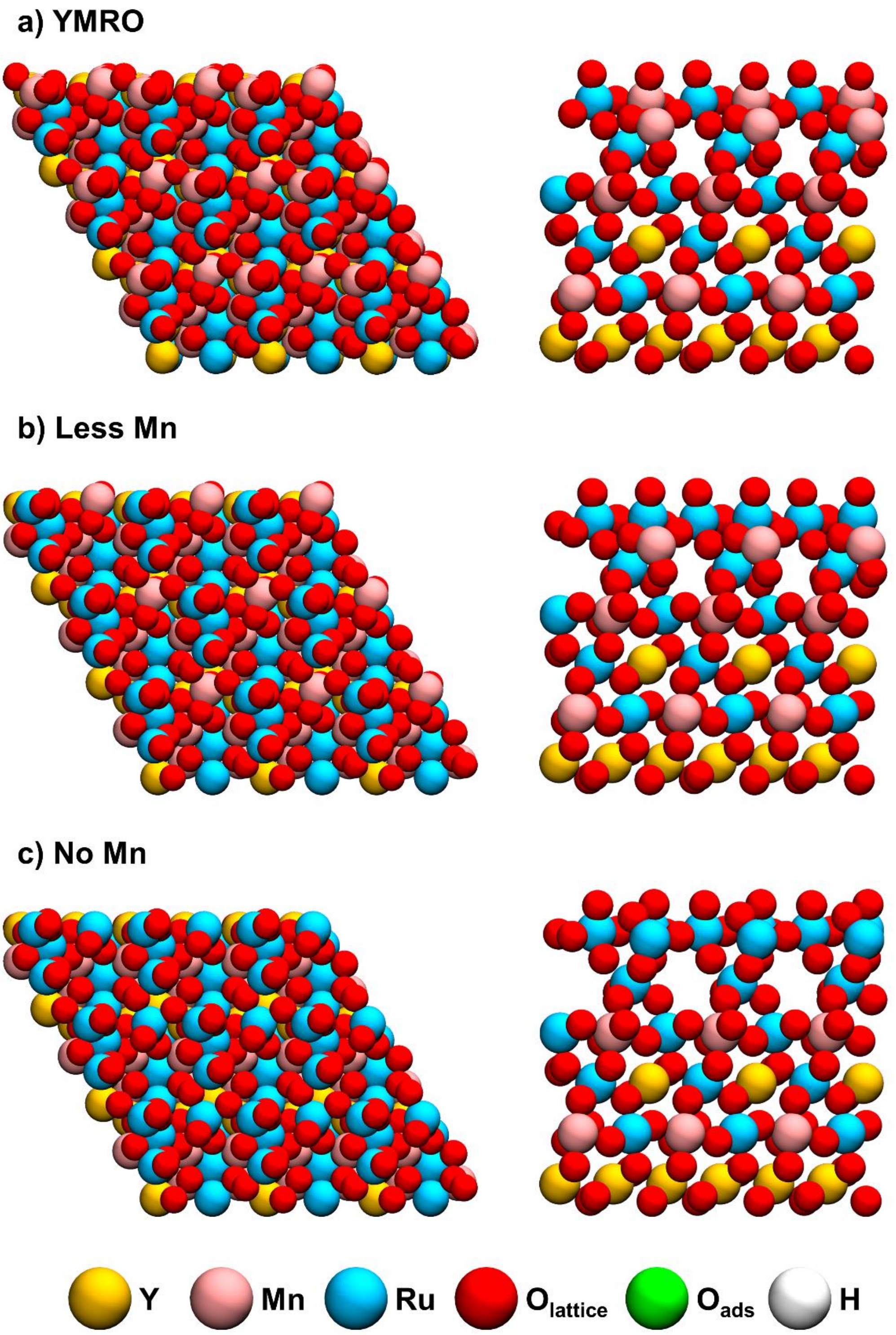

Figure S9. Top and side views of the relaxed slabs with various proportions of Mn to Ru in the topmost layers. a) YMRO with equal amounts of Ru and Mn on the top layer. b) YMRO with less Mn than Ru on the top layer. c) YMRO with no Mn on the top layer.

The Zero-point energies (ZPEs) and entropy corrections (TSs) of the molecules and adsorbates in this work are provided in Table S6 at T = 298.15 K. S includes all types of

entropies for molecules and vibrational contributions for adsorbates. The adsorption energies of *O, *OH and *OOH appear in Table S7.

Table S6. Zero-point energies and entropy corrections at 298.15 K for the free molecules and adsorbates in this study. All values are in eV.

species	ZPE	TS
**H_2O(l)**	0.57	0.67
**H_2(g)**	0.27	0.40
***OH**	0.35	0.10
***O**	0.08	0.05
***OOH**	0.44	0.19

Table S7. Adsorption energies of *OH, *O, and *OOH (in eV), OER overpotentials (in V) and electrochemical-step symmetry indices (ESSI, in V) of Y_2MnRuO_7 with a 1:1 ratio of Ru and Mn at surface (denoted as YMRO), with less Mn than Ru and with no Mn at the surface.

system	ΔG_{OH}	ΔG_{O}	ΔG_{OOH}	η_{OER}	ESSI
**YMRO**	1.29	2.79	4.32	0.30	0.21
**less Mn**	0.00	1.53	3.03	0.66	0.41
**no Mn**	1.98	3.73	4.69	0.75	0.64

The method to add the experimental datapoint of Y_2MnRuO_6 in Figure 6 is based on ref.[17] The method uses the experimental OER overpotential corresponding to a current density of 1 mA $cm^{-2}{}_{As}$, which for Y_2MnRuO_6 is around 0.29 V, according to Figure S2c. That overpotential is used to approximate $\Delta G_O - \Delta G_{OH}$ by means of the semiempirical volcano plot in ref.[17] In particular, 0.29 V corresponds to $\Delta G_O - \Delta G_{OH} \approx 1.53\ eV$, knowing that Y_2MnRuO_7 is on the strong-binding side of the volcano, as revealed by our DFT calculations (see Figure 6 in the main text).

S1.3 Supplementary Converged coordinates

YMRO, clean

```
  1.0000000000000000
    7.1831005684999996    0.0000000000000000    0.0000000000000000
   -3.5915502842999998    6.2436617694000001    0.0000000000000000
    0.0000000000000000    0.0000000000000000   35.0000000000000000
  Mn   Ru   Y    O
    6    6    8   40
Selective dynamics
Direct
  0.3342505465000016  0.1685010929000015  0.6843022441000031   F   F   F
  0.8342505465000016  0.1685010929000015  0.6843022441000031   F   F   F
  0.0019654566999989  0.5039309133999978  0.8523575713999989   F   F   F
  0.5019654566999989  0.5039309133999978  0.8523575713999989   F   F   F
  0.9125571546358690  0.9225835421903391  0.9674770107333652   T   T   T
  0.2541143148995630  0.8334135161291725  1.0133650622450773   T   T   T
  0.8342505465000016  0.6685010929000015  0.6843022441000031   F   F   F
  0.0003930912999976  0.0007861826999971  0.6002745803999971   F   F   F
  0.5019654566999989  0.0039309133999978  0.8523575713999989   F   F   F
  0.6681080015999967  0.3362160032000006  0.7683299077000001   F   F   F
  0.1339443673205790  0.3369737960435804  1.0109946642302048   T   T   T
  0.3168682161989712  0.6516591211868697  0.9303980687192248   T   T   T
  0.0003930912999976  0.5007861826999971  0.6002745803999971   F   F   F
  0.5003930912999976  0.5007861826999971  0.6002745803999971   F   F   F
  0.5003930912999976  0.0007861826999971  0.6002745803999971   F   F   F
  0.3342505465000016  0.6685010929000015  0.6843022441000031   F   F   F
  0.6681080015999967  0.8362160032000006  0.7683299077000001   F   F   F
  0.1681080015999967  0.8362160032000006  0.7683299077000001   F   F   F
  0.1681080015999967  0.3362160032000006  0.7683299077000001   F   F   F
  0.0019654566999989  0.0039309133999978  0.8523575713999989   F   F   F
  0.1432256068000015  0.2864512136999977  0.5733018912999981   F   F   F
  0.5323249409999988  0.0646498820999994  0.6712332314000022   F   F   F
  0.1404902625999966  0.8637678504000021  0.5714285713999985   F   F   F
  0.5264974505999973  0.4702075758999982  0.6709513950000030   F   F   F
  0.7232775877999984  0.8637678504000021  0.5714285713999985   F   F   F
  0.9437101253000009  0.4702075758999982  0.6709513950000030   F   F   F
  0.8575605759000027  0.7151211517000036  0.6272472695000033   F   F   F
  0.1361761519000027  0.2723523037999982  0.6973712567000021   F   F   F
  0.8602959201000004  0.1378045148999973  0.6291205894000029   F   F   F
  0.1420036423999989  0.8667946099999995  0.6976530931000013   F   F   F
  0.2775085947999969  0.1378045148999973  0.6291205894000029   F   F   F
  0.7247909676000006  0.8667946099999995  0.6976530931000013   F   F   F
  0.6662329541000034  0.3324659083000014  0.5790925471999984   F   F   F
  0.3345532286000008  0.6691064570999998  0.6214566136000030   F   F   F
  0.8109405171000006  0.6218810340999994  0.7413572186000010   F   F   F
  0.2000398512999979  0.4000797026000029  0.8392885586999981   F   F   F
  0.8082051728000010  0.1991976708999985  0.7394838987000014   F   F   F
  0.1942123608000017  0.8056373964000016  0.8390067222999988   F   F   F
  0.3909924980999975  0.1991976708999985  0.7394838987000014   F   F   F
  0.6114250355999999  0.8056373964000016  0.8390067222999988   F   F   F
  0.5252754861000000  0.0505509721999999  0.7953025967999992   F   F   F
  0.8038910620999999  0.6077821241999999  0.8654265839999979   F   F   F
  0.5280108302999977  0.4732343354000008  0.7971759166999988   F   F   F
  0.8097185526000032  0.2022244305000029  0.8657084203999972   F   F   F
  0.9452235051000031  0.4732343354000008  0.7971759166999988   F   F   F
  0.3925058777999979  0.2022244305000029  0.8657084203999972   F   F   F
  0.3339478644000025  0.6678957287999978  0.7471478745000013   F   F   F
  0.0022681387999981  0.0045362776000033  0.7895119408999989   F   F   F
  0.4367230256460103  0.9140401703452867  0.9061949791100485   T   T   T
  0.1399153160568972  0.9935525425447339  1.0013130766609928   T   T   T
  0.4778182151575404  0.5572245927585613  0.9036195580528468   T   T   T
  0.8725328202099607  0.1345048712032878  0.9900490102959711   T   T   T
  0.0605739595320545  0.5300611132325164  0.9129658465140805   T   T   T
  0.3084227247671535  0.4362291496307090  0.9699881664481718   T   T   T
  0.3683545065142406  0.8636835710122497  1.0533587024756915   T   T   T
  0.4078520510517154  0.8456118742126958  0.9738748846613534   T   T   T
  0.1910086728737436  0.3184986050379898  1.0569371875798793   T   T   T
  0.7339571781718250  0.6915748737972209  0.9811228861372995   T   T   T
  0.0501733750670160  0.5489817408509241  1.0132102224623453   T   T   T
  0.9851915501294486  0.9877070036900805  0.9225673549684548   T   T   T
```

YMRO + *O

```
  1.0000000000000000
    7.1831005684999996    0.0000000000000000    0.0000000000000000
   -3.5915502842999998    6.2436617694000001    0.0000000000000000
    0.0000000000000000    0.0000000000000000   35.0000000000000000
  Mn   Ru   Y    O
    6    6    8   41
Selective dynamics
Direct
  0.3342505465000016  0.1685010929000015  0.6843022441000031   F   F   F
  0.8342505465000016  0.1685010929000015  0.6843022441000031   F   F   F
  0.0019654566999989  0.5039309133999978  0.8523575713999989   F   F   F
  0.5019654566999989  0.5039309133999978  0.8523575713999989   F   F   F
  0.9441204441062989  0.9124173735977883  0.9666411654025163   T   T   T
  0.3243063287334043  0.9139755413412105  1.0134251164038541   T   T   T
  0.8342505465000016  0.6685010929000015  0.6843022441000031   F   F   F
  0.0003930912999976  0.0007861826999971  0.6002745803999971   F   F   F
  0.5019654566999989  0.0039309133999978  0.8523575713999989   F   F   F
  0.6681080015999967  0.3362160032000006  0.7683299077000001   F   F   F
  0.1867234759820830  0.4304886226489029  1.0268900750755351   T   T   T
  0.3061409509527403  0.6262104647242984  0.9336215131587986   T   T   T
  0.0003930912999976  0.5007861826999971  0.6002745803999971   F   F   F
  0.5003930912999976  0.5007861826999971  0.6002745803999971   F   F   F
  0.5003930912999976  0.0007861826999971  0.6002745803999971   F   F   F
  0.3342505465000016  0.6685010929000015  0.6843022441000031   F   F   F
  0.6681080015999967  0.8362160032000006  0.7683299077000001   F   F   F
  0.1681080015999967  0.8362160032000006  0.7683299077000001   F   F   F
  0.1681080015999967  0.3362160032000006  0.7683299077000001   F   F   F
  0.0019654566999989  0.0039309133999978  0.8523575713999989   F   F   F
  0.1432256068000015  0.2864512136999977  0.5733018912999981   F   F   F
  0.5323249409999988  0.0646498820999994  0.6712332314000022   F   F   F
  0.1404902625999966  0.8637678504000021  0.5714285713999985   F   F   F
  0.5264974505999973  0.4702075758999982  0.6709513950000030   F   F   F
  0.7232775877999984  0.8637678504000021  0.5714285713999985   F   F   F
  0.9437101253000009  0.4702075758999982  0.6709513950000030   F   F   F
  0.8575605759000027  0.7151211517000036  0.6272472695000033   F   F   F
  0.1361761519000027  0.2723523037999982  0.6973712567000021   F   F   F
  0.8602959201000004  0.1378045148999973  0.6291205894000029   F   F   F
  0.1420036423999989  0.8667946099999995  0.6976530931000013   F   F   F
  0.2775085947999969  0.1378045148999973  0.6291205894000029   F   F   F
  0.7247909676000006  0.8667946099999995  0.6976530931000013   F   F   F
  0.6662329541000034  0.3324659083000014  0.5790925471999984   F   F   F
  0.3345532286000008  0.6691064570999998  0.6214566136000030   F   F   F
  0.8109405171000006  0.6218810340999994  0.7413572186000010   F   F   F
  0.2000398512999979  0.4000797026000029  0.8392885586999981   F   F   F
  0.8082051728000010  0.1991976708999985  0.7394838987000014   F   F   F
  0.1942123608000017  0.8056373964000016  0.8390067222999988   F   F   F
  0.3909924980999975  0.1991976708999985  0.7394838987000014   F   F   F
  0.6114250355999999  0.8056373964000016  0.8390067222999988   F   F   F
  0.5252754861000000  0.0505509721999999  0.7953025967999992   F   F   F
  0.8038910620999999  0.6077821241999999  0.8654265839999979   F   F   F
  0.5280108302999977  0.4732343354000008  0.7971759166999988   F   F   F
  0.8097185526000032  0.2022244305000029  0.8657084203999972   F   F   F
  0.9452235051000031  0.4732343354000008  0.7971759166999988   F   F   F
  0.3925058777999979  0.2022244305000029  0.8657084203999972   F   F   F
  0.3339478644000025  0.6678957287999978  0.7471478745000013   F   F   F
  0.0022681387999981  0.0045362776000033  0.7895119408999989   F   F   F
  0.4486463521016701  0.8992415462090126  0.8999346829704777   T   T   T
  0.1482883623125482  0.0182875960618314  1.0000841150679018   T   T   T
  0.4830849319754140  0.5571115608987051  0.9127376219058887   T   T   T
  0.8033186810819219  0.0223140080429380  0.9736581012433342   T   T   T
  0.0607436522715477  0.5498121063414754  0.9116952769052080   T   T   T
  0.1904635321980542  0.4074501893884690  0.9726482656784609   T   T   T
  0.4795844408605996  0.0227680427610965  1.0486000063267914   T   T   T
  0.4051249650840830  0.8532263564087346  0.9713551871189107   T   T   T
  0.4390647831818861  0.5272057596405656  1.0437964062834435   T   T   T
  0.8040305278010611  0.6600514592955369  0.9732317489442358   T   T   T
  0.1233681816682774  0.6586979888687851  1.0289936317351623   T   T   T
  0.0394766953336670  0.9629832637663018  0.9231829026837716   T   T   T
  0.9528800334404974  0.2289635332034488  1.0442215269210933   T   T   T
```

YMRO + OH

```
  1.0000000000000000
    7.1831005684999996    0.0000000000000000    0.0000000000000000
   -3.5915502842999998    6.2436617694000001    0.0000000000000000
    0.0000000000000000    0.0000000000000000   35.0000000000000000
  Mn   Ru   Y    O    H
    6    6    8   41    1
Selective dynamics
Direct
  0.3342505465000016  0.1685010929000015  0.6843022441000031   F   F   F
  0.8342505465000016  0.1685010929000015  0.6843022441000031   F   F   F
  0.0019654566999989  0.5039309133999978  0.8523575713999989   F   F   F
  0.5019654566999989  0.5039309133999978  0.8523575713999989   F   F   F
  0.9457587540656054  0.9183046631400049  0.9682451005349311   T   T   T
  0.3215493764764285  0.8543468294651995  1.0097463605602245   T   T   T
  0.8342505465000016  0.6685010929000015  0.6843022441000031   F   F   F
  0.0003930912999976  0.0007861826999971  0.6002745803999971   F   F   F
  0.5019654566999989  0.0039309133999978  0.8523575713999989   F   F   F
  0.6681080015999967  0.3362160032000006  0.7683299077000001   F   F   F
  0.1048911710955020  0.3369387765306315  1.0235446219158095   T   T   T
  0.2962702008764402  0.5834399773722118  0.9337394697966946   T   T   T
  0.0003930912999976  0.5007861826999971  0.6002745803999971   F   F   F
  0.5003930912999976  0.5007861826999971  0.6002745803999971   F   F   F
  0.5003930912999976  0.0007861826999971  0.6002745803999971   F   F   F
  0.3342505465000016  0.6685010929000015  0.6843022441000031   F   F   F
  0.6681080015999967  0.8362160032000006  0.7683299077000001   F   F   F
  0.1681080015999967  0.8362160032000006  0.7683299077000001   F   F   F
  0.1681080015999967  0.3362160032000006  0.7683299077000001   F   F   F
  0.0019654566999989  0.0039309133999978  0.8523575713999989   F   F   F
  0.1432256068000015  0.2864512136999977  0.5733018912999981   F   F   F
  0.5323249409999988  0.0646498820999994  0.6712332314000022   F   F   F
  0.1404902625999966  0.8637678504000021  0.5714285713999985   F   F   F
  0.5264974505999973  0.4702075758999982  0.6709513950000030   F   F   F
  0.7232775877999984  0.8637678504000021  0.5714285713999985   F   F   F
  0.9437101253000009  0.4702075758999982  0.6709513950000030   F   F   F
  0.8575605759000027  0.7151211517000036  0.6272472695000033   F   F   F
  0.1361761519000027  0.2723523037999982  0.6973712567000021   F   F   F
  0.8602959201000004  0.1378045148999973  0.6291205894000029   F   F   F
  0.1420036423999989  0.8667946099999995  0.6976530931000013   F   F   F
  0.2775085947999969  0.1378045148999973  0.6291205894000029   F   F   F
  0.7247909676000006  0.8667946099999995  0.6976530931000013   F   F   F
  0.6662329541000034  0.3324659083000014  0.5790925471999984   F   F   F
  0.3345532286000008  0.6691064570999998  0.6214566136000030   F   F   F
  0.8109405171000006  0.6218810340999994  0.7413572186000010   F   F   F
  0.2000398512999979  0.4000797026000029  0.8392885586999981   F   F   F
  0.8082051728000010  0.1991976708999985  0.7394838987000014   F   F   F
  0.1942123608000017  0.8056373964000016  0.8390067222999988   F   F   F
  0.3909924980999975  0.1991976708999985  0.7394838987000014   F   F   F
  0.6114250355999999  0.8056373964000016  0.8390067222999988   F   F   F
  0.5252754861000000  0.0505509721999999  0.7953025967999992   F   F   F
  0.8038910620999999  0.6077821241999999  0.8654265839999979   F   F   F
  0.5280108302999977  0.4732343354000008  0.7971759166999988   F   F   F
  0.8097185526000032  0.2022244305000029  0.8657084203999972   F   F   F
  0.9452235051000031  0.4732343354000008  0.7971759166999988   F   F   F
  0.3925058777999979  0.2022244305000029  0.8657084203999972   F   F   F
  0.3339478644000025  0.6678957287999978  0.7471478745000013   F   F   F
  0.0022681387999981  0.0045362776000033  0.7895119408999989   F   F   F
  0.4485349578433826  0.8914825240581233  0.8974772794080041   T   T   T
  0.2035677354018296  0.9917923771223883  0.9902352471114423   T   T   T
  0.4817964422279732  0.5301094433910560  0.9112952266645468   T   T   T
  0.8872444557907885  0.0720698956518758  1.0027773608359324   T   T   T
  0.0631572448137863  0.5498679116604617  0.9123864436150040   T   T   T
  0.1833991857743405  0.3580629390554045  0.9676998008072966   T   T   T
  0.4447460409836990  0.9349023224092482  1.0493462150028924   T   T   T
  0.4423442172208014  0.8036918331988364  0.9700293565097979   T   T   T
  0.3153300888454208  0.3533564042371642  1.0465623755538997   T   T   T
  0.7919776178314938  0.6747691158721432  0.9795078224154503   T   T   T
  0.0953693963130234  0.6001494776148614  1.0198361756999424   T   T   T
  0.9700799349566586  0.9853927561729475  0.9227241222210880   T   T   T
  0.8988304813135337  0.2845301020824761  1.0621477741648369   T   T   T
  0.8071434694652920  0.1285440576505517  1.0650868088368778   T   T   T
```

YMRO + OOH

```
  1.0000000000000000
    7.1831005684999996    0.0000000000000000    0.0000000000000000
   -3.5915502842999998    6.2436617694000001    0.0000000000000000
    0.0000000000000000    0.0000000000000000   35.0000000000000000
  Mn   Ru   Y    O    H
    6    6    8   42    1
Selective dynamics
Direct
  0.3342505465000016  0.1685010929000015  0.6843022441000031   F   F   F
  0.8342505465000016  0.1685010929000015  0.6843022441000031   F   F   F
  0.0019654566999989  0.5039309133999978  0.8523575713999989   F   F   F
  0.5019654566999989  0.5039309133999978  0.8523575713999989   F   F   F
  0.0716710901198423  0.0949646379915033  0.9680539263694744   T   T   T
  0.3403439919111381  0.9028280744579279  1.0116900055014293   T   T   T
  0.8342505465000016  0.6685010929000015  0.6843022441000031   F   F   F
  0.0003930912999976  0.0007861826999971  0.6002745803999971   F   F   F
  0.5019654566999989  0.0039309133999978  0.8523575713999989   F   F   F
  0.6681080015999967  0.3362160032000006  0.7683299077000001   F   F   F
 -0.0198220982470854  0.6492106123675500  1.0784452697496874   T   T   T
  0.2943795913569919  0.5886213869928622  0.9341429597647368   T   T   T
  0.0003930912999976  0.5007861826999971  0.6002745803999971   F   F   F
  0.5003930912999976  0.5007861826999971  0.6002745803999971   F   F   F
  0.5003930912999976  0.0007861826999971  0.6002745803999971   F   F   F
  0.3342505465000016  0.6685010929000015  0.6843022441000031   F   F   F
  0.6681080015999967  0.8362160032000006  0.7683299077000001   F   F   F
  0.1681080015999967  0.8362160032000006  0.7683299077000001   F   F   F
  0.1681080015999967  0.3362160032000006  0.7683299077000001   F   F   F
  0.0019654566999989  0.0039309133999978  0.8523575713999989   F   F   F
  0.1432256068000015  0.2864512136999977  0.5733018912999981   F   F   F
  0.5323249409999988  0.0646498820999994  0.6712332314000022   F   F   F
  0.1404902625999966  0.8637678504000021  0.5714285713999985   F   F   F
  0.5264974505999973  0.4702075758999982  0.6709513950000030   F   F   F
  0.7232775877999984  0.8637678504000021  0.5714285713999985   F   F   F
  0.9437101253000009  0.4702075758999982  0.6709513950000030   F   F   F
  0.8575605759000027  0.7151211517000036  0.6272472695000033   F   F   F
  0.1361761519000027  0.2723523037999982  0.6973712567000021   F   F   F
  0.8602959201000004  0.1378045148999973  0.6291205894000029   F   F   F
  0.1420036423999989  0.8667946099999995  0.6976530931000013   F   F   F
  0.2775085947999969  0.1378045148999973  0.6291205894000029   F   F   F
  0.7247909676000006  0.8667946099999995  0.6976530931000013   F   F   F
  0.6662329541000034  0.3324659083000014  0.5790925471999984   F   F   F
  0.3345532286000008  0.6691064570999998  0.6214566136000030   F   F   F
  0.8109405171000006  0.6218810340999994  0.7413572186000010   F   F   F
  0.2000398512999979  0.4000797026000029  0.8392885586999981   F   F   F
  0.8082051728000010  0.1991976708999985  0.7394838987000014   F   F   F
  0.1942123608000017  0.8056373964000016  0.8390067222999988   F   F   F
  0.3909924980999975  0.1991976708999985  0.7394838987000014   F   F   F
  0.6114250355999999  0.8056373964000016  0.8390067222999988   F   F   F
  0.5252754861000000  0.0505509721999999  0.7953025967999992   F   F   F
  0.8038910620999999  0.6077821241999999  0.8654265839999979   F   F   F
  0.5280108302999977  0.4732343354000008  0.7971759166999988   F   F   F
  0.8097185526000032  0.2022244305000029  0.8657084203999972   F   F   F
  0.9452235051000031  0.4732343354000008  0.7971759166999988   F   F   F
  0.3925058777999979  0.2022244305000029  0.8657084203999972   F   F   F
  0.3339478644000025  0.6678957287999978  0.7471478745000013   F   F   F
  0.0022681387999981  0.0045362776000033  0.7895119408999989   F   F   F
  0.4412407415538552  0.8863426805639211  0.8972034210824225   T   T   T
  0.2892887482673593  0.1029574090750043  1.0010142783018895   T   T   T
  0.4845623175037470  0.5385098868096722  0.9118929355657872   T   T   T
  0.7319602430757646  0.4288139265655024  1.0752844437996240   T   T   T
  0.0529025330531493  0.5276555194880873  0.9116677619083807   T   T   T
  0.2025470702513429  0.3873544206191547  0.9728665575749701   T   T   T
  0.5198366625502188  0.9522524315678170  1.0430751322663744   T   T   T
  0.4073141571861880  0.8238488529172873  0.9682969025829223   T   T   T
  0.1484459771547801  0.5604835896039488  1.1060316130483770   T   T   T
  0.8622171513904090 -0.0094546529661192  0.9939123328217021   T   T   T
  0.1044119926774442  0.6761695463054540  1.0282284666978950   T   T   T
  0.0758462490444747  0.0382015106652412  0.9228011341136949   T   T   T
  0.0485008855470235  0.8992099028335866  1.0934453782371988   T   T   T
  0.3007179927733988  0.5237113018756604  1.0860234538913216   T   T   T
  0.4382179462821520  0.6434465779398097  1.0952820881385902   T   T   T
```

less Mn, clean

```
  1.0000000000000000
    7.1831005684999996    0.0000000000000000    0.0000000000000000
   -3.5915502842999998    6.2436617694000001    0.0000000000000000
    0.0000000000000000    0.0000000000000000   35.0000000000000000
  Mn   Ru   Y    O
    5    7    8   40
Selective dynamics
```

Direct
0.3342505465000016 0.1685010929000015 0.6843022441000031 F F F
0.8342505465000016 0.1685010929000015 0.6843022441000031 F F F
0.0019654566999989 0.5039309133999978 0.8523575713999989 F F F
0.5019654566999989 0.5039309133999978 0.8523575713999989 F F F
0.9086973861936851 0.9216745158962569 0.9702960700220603 T T T
0.2740618340407204 0.8456365277138839 0.0177930211191886 T T T
0.8342505465000016 0.6685010929000015 0.6843022441000031 F F F
0.0003930912999976 0.0007861826999971 0.6002745803999971 F F F
0.5019654566999989 0.0039309133999978 0.8523575713999989 F F F
0.6681080015999967 0.3362160032000006 0.7683299077000001 F F F
0.1348719586351434 0.3338542474732143 0.0136651567672188 T T T
0.3219751881818782 0.6519232865184275 0.9313764428208317 T T T
0.0003930912999976 0.5007861826999971 0.6002745803999971 F F F
0.5003930912999976 0.5007861826999971 0.6002745803999971 F F F
0.5003930912999976 0.0007861826999971 0.6002745803999971 F F F
0.3342505465000016 0.6685010929000015 0.6843022441000031 F F F
0.6681080015999967 0.8362160032000006 0.7683299077000001 F F F
0.1681080015999967 0.8362160032000006 0.7683299077000001 F F F
0.1681080015999967 0.3362160032000006 0.7683299077000001 F F F
0.0019654566999989 0.0039309133999978 0.8523575713999989 F F F
0.1432256068000015 0.2864512136999977 0.5733018912999981 F F F
0.5323249409999988 0.0646498820999994 0.6712332314000022 F F F
0.1404902625999966 0.8637678504000021 0.5714285713999985 F F F
0.5264974505999973 0.4702075758999982 0.6709513950000030 F F F
0.7232775877999984 0.8637678504000021 0.5714285713999985 F F F
0.9437101253000009 0.4702075758999982 0.6709513950000030 F F F
0.8575605759000027 0.7151211517000036 0.6272472695000033 F F F
0.1361761519000027 0.2723523037999982 0.6973712567000021 F F F
0.8602959201000004 0.1378045148999973 0.6291205894000029 F F F
0.1420036423999989 0.8667946099999995 0.6976530931000013 F F F
0.2775085947999969 0.1378045148999973 0.6291205894000029 F F F
0.7247909676000006 0.8667946099999995 0.6976530931000013 F F F
0.6662329541000034 0.3324659083000014 0.5790925471999984 F F F
0.3345532286000008 0.6691064570999998 0.6214566136000030 F F F
0.8109405171000006 0.6218810340999994 0.7413572186000010 F F F
0.2000398512999979 0.4000797026000029 0.8392885586999981 F F F
0.8082051728000010 0.1991976708999985 0.7394838987000014 F F F
0.1942123608000017 0.8056373964000016 0.8390067222999988 F F F
0.3909924980999975 0.1991976708999985 0.7394838987000014 F F F
0.6114250355999999 0.8056373964000016 0.8390067222999988 F F F
0.5252754861000000 0.0505509721999999 0.7953025967999992 F F F
0.8038910620999999 0.6077821241999999 0.8654265839999979 F F F
0.5280108302999977 0.4732343354000008 0.7971759166999988 F F F
0.8097185526000032 0.2022244305000029 0.8657084203999972 F F F
0.9452235051000031 0.4732343354000008 0.7971759166999988 F F F
0.3925058777999979 0.2022244305000029 0.8657084203999972 F F F
0.3339478644000025 0.6678957287999978 0.7471478745000013 F F F
0.0022681387999981 0.0045362776000033 0.7895119408999989 F F F
0.4429749253755413 0.9126579379276095 0.9067574144450549 T T T
0.1353075217181833 0.0002166551694822 0.0048444339877572 T T T
0.4711915896527533 0.5490976797196121 0.9055970466756281 T T T
0.8692945771040429 0.1335369302817049 0.9933590734736458 T T T
0.0633696204495090 0.5337908439342959 0.9153431452709273 T T T
0.3039193545256176 0.4304197587071096 0.9723605819139567 T T T
0.3789935703763415 0.8667232811333773 0.0619070308811837 T T T
0.4240750500885806 0.8592930037119979 0.9747999999499057 T T T
0.1893449870219240 0.3165900251311948 0.0601150568976275 T T T
0.7326185953472654 0.6887739217002715 0.9838357445800797 T T T
0.0533151977975671 0.5513715743510426 0.0156113727783937 T T T
0.9796458280924148 0.9844853893042644 0.9255377616281548 T T T

less Mn + O
1.0000000000000000
7.1831005684999996 0.0000000000000000 0.0000000000000000
-3.5915502842999998 6.2436617694000001 0.0000000000000000
0.0000000000000000 0.0000000000000000 35.0000000000000000
Mn Ru Y O
5 7 8 41
Selective dynamics
Direct
0.3342505465000016 0.1685010929000015 0.6843022441000031 F F F
0.8342505465000016 0.1685010929000015 0.6843022441000031 F F F
0.0019654566999989 0.5039309133999978 0.8523575713999989 F F F
0.5019654566999989 0.5039309133999978 0.8523575713999989 F F F
0.9337176710433472 0.8994047617812496 0.9652105103693538 T T T
0.3366323304016408 0.9312250292177043 0.0173152460308196 T T T
0.8342505465000016 0.6685010929000015 0.6843022441000031 F F F
0.0003930912999976 0.0007861826999971 0.6002745803999971 F F F
0.5019654566999989 0.0039309133999978 0.8523575713999989 F F F
0.6681080015999967 0.3362160032000006 0.7683299077000001 F F F
0.1929510817459916 0.4214662454085986 0.0277099494463677 T T T
0.2992918637808777 0.6211390015374715 0.9347404706078351 T T T
0.0003930912999976 0.5007861826999971 0.6002745803999971 F F F
0.5003930912999976 0.5007861826999971 0.6002745803999971 F F F
0.5003930912999976 0.0007861826999971 0.6002745803999971 F F F
0.3342505465000016 0.6685010929000015 0.6843022441000031 F F F
0.6681080015999967 0.8362160032000006 0.7683299077000001 F F F
0.1681080015999967 0.8362160032000006 0.7683299077000001 F F F
0.1681080015999967 0.3362160032000006 0.7683299077000001 F F F
0.0019654566999989 0.0039309133999978 0.8523575713999989 F F F
0.1432256068000015 0.2864512136999977 0.5733018912999981 F F F
0.5323249409999988 0.0646498820999994 0.6712332314000022 F F F
0.1404902625999966 0.8637678504000021 0.5714285713999985 F F F
0.5264974505999973 0.4702075758999982 0.6709513950000030 F F F
0.7232775877999984 0.8637678504000021 0.5714285713999985 F F F
0.9437101253000009 0.4702075758999982 0.6709513950000030 F F F
0.8575605759000027 0.7151211517000036 0.6272472695000033 F F F
0.1361761519000027 0.2723523037999982 0.6973712567000021 F F F
0.8602959201000004 0.1378045148999973 0.6291205894000029 F F F
0.1420036423999989 0.8667946099999995 0.6976530931000013 F F F
0.2775085947999969 0.1378045148999973 0.6291205894000029 F F F
0.7247909676000006 0.8667946099999995 0.6976530931000013 F F F
0.6662329541000034 0.3324659083000014 0.5790925471999984 F F F
0.3345532286000008 0.6691064570999998 0.6214566136000030 F F F
0.8109405171000006 0.6218810340999994 0.7413572186000010 F F F
0.2000398512999979 0.4000797026000029 0.8392885586999981 F F F
0.8082051728000010 0.1991976708999985 0.7394838987000014 F F F
0.1942123608000017 0.8056373964000016 0.8390067222999988 F F F
0.3909924980999975 0.1991976708999985 0.7394838987000014 F F F
0.6114250355999999 0.8056373964000016 0.8390067222999988 F F F
0.5252754861000000 0.0505509721999999 0.7953025967999992 F F F
0.8038910620999999 0.6077821241999999 0.8654265839999979 F F F
0.5280108302999977 0.4732343354000008 0.7971759166999988 F F F
0.8097185526000032 0.2022244305000029 0.8657084203999972 F F F
0.9452235051000031 0.4732343354000008 0.7971759166999988 F F F
0.3925058777999979 0.2022244305000029 0.8657084203999972 F F F
0.3339478644000025 0.6678957287999978 0.7471478745000013 F F F
0.0022681387999981 0.0045362776000033 0.7895119408999989 F F F
0.4454855368144831 0.8878212983886702 0.8975388403618449 T T T
0.1256566357886892 0.0162534957792249 0.9994093758887649 T T T
0.4604089854404917 0.5365170702653258 0.9119231159049511 T T T
0.7568151618783457 0.9641036755645149 0.9747936331300375 T T T
0.0486937672497927 0.5091415260970759 0.9069495048376409 T T T
0.1491491973037350 0.4198217291547641 0.9740223335510844 T T T
0.5441860084720824 0.0998957419982916 0.0449395391632506 T T T
0.3530689438769460 0.8522940172897636 0.9647623544050409 T T T
0.4523093226674562 0.4978327509406170 0.0373134700437359 T T T
0.8315295397607309 0.6462202394236410 0.9681345598300856 T T T
0.1553251327506498 0.6576994731189478 0.0390929811728184 T T T
0.0253086982208671 0.9809599253650165 0.9216608708359043 T T T
0.9728900260769490 0.2232065611933080 0.0494038602598392 T T T

less Mn + OH
1.0000000000000000
7.1831005684999996 0.0000000000000000 0.0000000000000000
-3.5915502842999998 6.2436617694000001 0.0000000000000000
0.0000000000000000 0.0000000000000000 35.0000000000000000
Mn Ru Y O H
5 7 8 41 1
Selective dynamics
Direct
0.3342505465000016 0.1685010929000015 0.6843022441000031 F F F
0.8342505465000016 0.1685010929000015 0.6843022441000031 F F F
0.0019654566999989 0.5039309133999978 0.8523575713999989 F F F
0.5019654566999989 0.5039309133999978 0.8523575713999989 F F F
0.9592171587686676 0.9355115572939497 0.9672199031435192 T T T
0.3551549949890707 0.8910077370376728 0.0105287105305945 T T T
0.8342505465000016 0.6685010929000015 0.6843022441000031 F F F
0.0003930912999976 0.0007861826999971 0.6002745803999971 F F F
0.5019654566999989 0.0039309133999978 0.8523575713999989 F F F
0.6681080015999967 0.3362160032000006 0.7683299077000001 F F F
0.1243165608474144 0.3602897911879835 0.0212572369568808 T T T
0.3112385452792192 0.6137842061443308 0.9317799143641320 T T T
0.0003930912999976 0.5007861826999971 0.6002745803999971 F F F
0.5003930912999976 0.5007861826999971 0.6002745803999971 F F F
0.5003930912999976 0.0007861826999971 0.6002745803999971 F F F
0.3342505465000016 0.6685010929000015 0.6843022441000031 F F F
0.6681080015999967 0.8362160032000006 0.7683299077000001 F F F
0.1681080015999967 0.8362160032000006 0.7683299077000001 F F F
0.1681080015999967 0.3362160032000006 0.7683299077000001 F F F
0.0019654566999989 0.0039309133999978 0.8523575713999989 F F F
0.1432256068000015 0.2864512136999977 0.5733018912999981 F F F
0.5323249409999988 0.0646498820999994 0.6712332314000022 F F F
0.1404902625999966 0.8637678504000021 0.5714285713999985 F F F
0.5264974505999973 0.4702075758999982 0.6709513950000030 F F F
0.7232775877999984 0.8637678504000021 0.5714285713999985 F F F
0.9437101253000009 0.4702075758999982 0.6709513950000030 F F F
0.8575605759000027 0.7151211517000036 0.6272472695000033 F F F
0.1361761519000027 0.2723523037999982 0.6973712567000021 F F F
0.8602959201000004 0.1378045148999973 0.6291205894000029 F F F
0.1420036423999989 0.8667946099999995 0.6976530931000013 F F F
0.2775085947999969 0.1378045148999973 0.6291205894000029 F F F
0.7247909676000006 0.8667946099999995 0.6976530931000013 F F F
0.6662329541000034 0.3324659083000014 0.5790925471999984 F F F
0.3345532286000008 0.6691064570999998 0.6214566136000030 F F F
0.8109405171000006 0.6218810340999994 0.7413572186000010 F F F
0.2000398512999979 0.4000797026000029 0.8392885586999981 F F F
0.8082051728000010 0.1991976708999985 0.7394838987000014 F F F
0.1942123608000017 0.8056373964000016 0.8390067222999988 F F F
0.3909924980999975 0.1991976708999985 0.7394838987000014 F F F
0.6114250355999999 0.8056373964000016 0.8390067222999988 F F F
0.5252754861000000 0.0505509721999999 0.7953025967999992 F F F
0.8038910620999999 0.6077821241999999 0.8654265839999979 F F F
0.5280108302999977 0.4732343354000008 0.7971759166999988 F F F
0.8097185526000032 0.2022244305000029 0.8657084203999972 F F F
0.9452235051000031 0.4732343354000008 0.7971759166999988 F F F
0.3925058777999979 0.2022244305000029 0.8657084203999972 F F F
0.3339478644000025 0.6678957287999978 0.7471478745000013 F F F
0.0022681387999981 0.0045362776000033 0.7895119408999989 F F F
0.4483076974196150 0.8988983936567275 0.8997878391787286 T T T
0.2341809617132413 0.0292034389078917 0.9874001194424239 T T T
0.4846885046215268 0.5437385426119310 0.9085830547248497 T T T
0.9100636556846154 0.0879489212774394 0.0052566557600276 T T T
0.0674944452647254 0.5540983195628727 0.9105713484104753 T T T
0.1943016559146934 0.3768595541580289 0.9668215512261246 T T T
0.4524414214081480 0.9680507507459658 0.0550552407090225 T T T
0.4790757184811984 0.8267463075721192 0.9711172116182867 T T T
0.3329958198802317 0.3754848250297106 0.0449388465852339 T T T
0.8137426924966948 0.6924437001797895 0.9798199658431579 T T T
0.1122086020838274 0.6287216619110999 0.0159830834027926 T T T
0.9723570521015663 0.0018090463516379 0.9221936068459096 T T T
0.9182491591362220 0.3217400775443499 0.0596771787323715 T T T
0.8233642331166127 0.1667116952383512 0.0630544997292973 T T T

less Mn + OOH
1.0000000000000000
7.1831005684999996 0.0000000000000000 0.0000000000000000
-3.5915502842999998 6.2436617694000001 0.0000000000000000
0.0000000000000000 0.0000000000000000 35.0000000000000000
Mn Ru Y O H
5 7 8 42 1
Selective dynamics
Direct
0.3342505465000016 0.1685010929000015 0.6843022441000031 F F F
0.8342505465000016 0.1685010929000015 0.6843022441000031 F F F
0.0019654566999989 0.5039309133999978 0.8523575713999989 F F F
0.5019654566999989 0.5039309133999978 0.8523575713999989 F F F
0.0291781891156355 0.1003083771239216 0.9661810179615147 T T T
0.2827244094235963 0.8797371735560150 0.0165848350185821 T T T
0.8342505465000016 0.6685010929000015 0.6843022441000031 F F F
0.0003930912999976 0.0007861826999971 0.6002745803999971 F F F
0.5019654566999989 0.0039309133999978 0.8523575713999989 F F F
0.6681080015999967 0.3362160032000006 0.7683299077000001 F F F
0.0115155871320536 0.5243968518550594 0.0873498585263506 T T T
0.2951076710445955 0.5975323520632742 0.9343559714978479 T T T
0.0003930912999976 0.5007861826999971 0.6002745803999971 F F F
0.5003930912999976 0.5007861826999971 0.6002745803999971 F F F
0.5003930912999976 0.0007861826999971 0.6002745803999971 F F F
0.3342505465000016 0.6685010929000015 0.6843022441000031 F F F
0.6681080015999967 0.8362160032000006 0.7683299077000001 F F F
0.1681080015999967 0.8362160032000006 0.7683299077000001 F F F
0.1681080015999967 0.3362160032000006 0.7683299077000001 F F F
0.0019654566999989 0.0039309133999978 0.8523575713999989 F F F
0.1432256068000015 0.2864512136999977 0.5733018912999981 F F F
0.5323249409999988 0.0646498820999994 0.6712332314000022 F F F
0.1404902625999966 0.8637678504000021 0.5714285713999985 F F F
0.5264974505999973 0.4702075758999982 0.6709513950000030 F F F
0.7232775877999984 0.8637678504000021 0.5714285713999985 F F F
0.9437101253000009 0.4702075758999982 0.6709513950000030 F F F
0.8575605759000027 0.7151211517000036 0.6272472695000033 F F F
0.1361761519000027 0.2723523037999982 0.6973712567000021 F F F
0.8602959201000004 0.1378045148999973 0.6291205894000029 F F F
0.1420036423999989 0.8667946099999995 0.6976530931000013 F F F
0.2775085947999969 0.1378045148999973 0.6291205894000029 F F F
0.7247909676000006 0.8667946099999995 0.6976530931000013 F F F
0.6662329541000034 0.3324659083000014 0.5790925471999984 F F F
0.3345532286000008 0.6691064570999998 0.6214566136000030 F F F
0.8109405171000006 0.6218810340999994 0.7413572186000010 F F F
0.2000398512999979 0.4000797026000029 0.8392885586999981 F F F
0.8082051728000010 0.1991976708999985 0.7394838987000014 F F F
0.1942123608000017 0.8056373964000016 0.8390067222999988 F F F
0.3909924980999975 0.1991976708999985 0.7394838987000014 F F F
0.6114250355999999 0.8056373964000016 0.8390067222999988 F F F
0.5252754861000000 0.0505509721999999 0.7953025967999992 F F F
0.8038910620999999 0.6077821241999999 0.8654265839999979 F F F
0.5280108302999977 0.4732343354000008 0.7971759166999988 F F F
0.8097185526000032 0.2022244305000029 0.8657084203999972 F F F
0.9452235051000031 0.4732343354000008 0.7971759166999988 F F F
0.3925058777999979 0.2022244305000029 0.8657084203999972 F F F
0.3339478644000025 0.6678957287999978 0.7471478745000013 F F F
0.0022681387999981 0.0045362776000033 0.7895119408999989 F F F
0.4421163705060532 0.8895447797351628 0.8978235757640498 T T T
0.1838497891337930 0.0618714842145688 0.0066101958889621 T T T
0.4917729121126363 0.5568068378184835 0.9134338109828193 T T T
0.8550612644091322 0.2541771939953553 0.0909070071594308 T T T
0.0523629413142684 0.5226597934563127 0.9112214533586147 T T T
0.2044628623292226 0.3904397673637008 0.9728068638677688 T T T
0.5104269248573686 0.9711448835787522 0.0424764906799284 T T T
0.3580772531094992 0.8325345452933319 0.9680733425422062 T T T
0.2801184824901082 0.5694226613677608 0.1050119153626763 T T T
0.8016635204535151 0.0443138962524114 0.9831521884552950 T T T
0.0769211979367667 0.6060248971980265 0.0342425834500775 T T T
0.0630519248665750 0.0356123556847540 0.9229349414331283 T T T
0.9677109196096886 0.7145400192875108 0.1073258036215570 T T T
0.4198417488730546 0.5571670405514680 0.0771130685469947 T T T
0.5458262082485571 0.7025156708040188 0.0783262717877602 T T T

no Mn, clean
1.0000000000000000
7.1831005684999996 0.0000000000000000 0.0000000000000000
-3.5915502842999998 6.2436617694000001 0.0000000000000000
0.0000000000000000 0.0000000000000000 35.0000000000000000
Mn Ru Y O
4 8 8 40
Selective dynamics
Direct
0.3342505465000016 0.1685010929000015 0.6843022441000031 F F F
0.8342505465000016 0.1685010929000015 0.6843022441000031 F F F
0.0019654566999989 0.5039309133999978 0.8523575713999989 F F F
0.5019654566999989 0.5039309133999978 0.8523575713999989 F F F
0.8431060323948139 0.8426326066760056 0.0081495850812645 T T T
0.3023185901434628 0.8393425132002709 0.0244658953940797 T T T
0.8342505465000016 0.6685010929000015 0.6843022441000031 F F F
0.0003930912999976 0.0007861826999971 0.6002745803999971 F F F
0.5019654566999989 0.0039309133999978 0.8523575713999989 F F F
0.6681080015999967 0.3362160032000006 0.7683299077000001 F F F
0.1505401344244111 0.3374625589030396 0.0207585573316047 T T T
0.3278594669469860 0.6590647958667130 0.9333415469227858 T T T
0.0003930912999976 0.5007861826999971 0.6002745803999971 F F F
0.5003930912999976 0.5007861826999971 0.6002745803999971 F F F
0.5003930912999976 0.0007861826999971 0.6002745803999971 F F F
0.3342505465000016 0.6685010929000015 0.6843022441000031 F F F

```
0.6681080015999967 0.8362160032000006 0.7683299077000001 F F F
0.1681080015999967 0.8362160032000006 0.7683299077000001 F F F
0.1681080015999967 0.3362160032000006 0.7683299077000001 F F F
0.0019654566999989 0.0039309133999978 0.8523575713999989 F F F
0.1432256068000015 0.2864512136999977 0.5733018912999981 F F F
0.5323249409999988 0.0646498820999994 0.6712332314000022 F F F
0.1404902625999966 0.8637678504000021 0.5714285713999985 F F F
0.5264974505999973 0.4702075758999982 0.6709513950000030 F F F
0.7232775877999984 0.8637678504000021 0.5714285713999985 F F F
0.9437101253000009 0.4702075758999982 0.6709513950000030 F F F
0.8575605759000027 0.7151211517000036 0.6272472695000033 F F F
0.1361761519000027 0.2723523037999982 0.6973712567000021 F F F
0.8602959201000004 0.1378045148999973 0.6291205894000029 F F F
0.1420036423999989 0.8667946099999995 0.6976530931000013 F F F
0.2775085947999969 0.1378045148999973 0.6291205894000029 F F F
0.7247909676000006 0.8667946099999995 0.6976530931000013 F F F
0.6662329541000034 0.3324659083000014 0.5790925471999984 F F F
0.3345532286000008 0.6691064570999998 0.6214566136000030 F F F
0.8109405171000006 0.6218810340999994 0.7413572186000010 F F F
0.2000398512999979 0.4000797026000029 0.8392885586999981 F F F
0.8082051728000010 0.1991976708999985 0.7394838987000014 F F F
0.1942123608000017 0.8056373964000016 0.8390067222999988 F F F
0.3909924980999975 0.1991976708999985 0.7394838987000014 F F F
0.6114250355999999 0.8056373964000016 0.8390067222999988 F F F
0.5252754861000000 0.0505509721999999 0.7953025967999992 F F F
0.8038910620999999 0.6077821241999999 0.8654265839999979 F F F
0.5280108302999977 0.4732343354000008 0.7971759166999988 F F F
0.8097185526000032 0.2022244305000029 0.8657084203999972 F F F
0.9452235051000031 0.4732343354000008 0.7971759166999988 F F F
0.3925058777999979 0.2022244305000029 0.8657084203999972 F F F
0.3339478644000025 0.6678957287999978 0.7471478745000013 F F F
0.0022681387999981 0.0045362776000033 0.7895119408999989 F F F
0.4493183478546026 0.9169828094152345 0.9079317442194138 T T T
0.1424276515583416 -0.0097855667218400 0.0177517540654214 T T T
0.4660545528588010 0.5486387793431472 0.9072020700176298 T T T
0.8609473154134455 0.1164011124917992 0.0135640098916792 T T T
0.0639449447933352 0.5497705069325559 0.9204539367245910 T T T
0.2860478972508973 0.4125528940410907 0.9762231988186675 T T T
0.4347110536893597 0.8815951322210823 0.0667949973396908 T T T
0.4367716183672843 0.8568806484005809 0.9770374424102090 T T T
0.2494534331301504 0.3547384139008471 0.0651216197805144 T T T
0.7109048148569613 0.6847057229418325 0.0458406794718956 T T T
0.0629225698381217 0.5451880015014184 0.0222714465889209 T T T
0.8186097687243390 0.7698068001820532 0.9615045902740234 T T T
```

no Mn + O

```
  1.0000000000000000
    7.1831005684999996    0.0000000000000000    0.0000000000000000
   -3.5915502842999998    6.2436617694000001    0.0000000000000000
    0.0000000000000000    0.0000000000000000   35.0000000000000000
  Mn   Ru   Y    O
    4    8    8   41
Selective dynamics
Direct
0.3342505465000016 0.1685010929000015 0.6843022441000031 F F F
0.8342505465000016 0.1685010929000015 0.6843022441000031 F F F
0.0019654566999989 0.5039309133999978 0.8523575713999989 F F F
0.5019654566999989 0.5039309133999978 0.8523575713999989 F F F
0.9391586524172317 0.8911399986993721 0.9702978512830491 T T T
0.3609820137667812 0.9209004009429463 0.0210051531240986 T T T
0.8342505465000016 0.6685010929000015 0.6843022441000031 F F F
0.0003930912999976 0.0007861826999971 0.6002745803999971 F F F
0.5019654566999989 0.0039309133999978 0.8523575713999989 F F F
0.6681080015999967 0.3362160032000006 0.7683299077000001 F F F
0.2169168126032613 0.4172995397555487 0.0327842344084912 T T T
0.2835523215301742 0.5977183390540682 0.9381559466919800 T T T
0.0003930912999976 0.5007861826999971 0.6002745803999971 F F F
0.5003930912999976 0.5007861826999971 0.6002745803999971 F F F
0.5003930912999976 0.0007861826999971 0.6002745803999971 F F F
0.3342505465000016 0.6685010929000015 0.6843022441000031 F F F
0.6681080015999967 0.8362160032000006 0.7683299077000001 F F F
0.1681080015999967 0.8362160032000006 0.7683299077000001 F F F
0.1681080015999967 0.3362160032000006 0.7683299077000001 F F F
0.0019654566999989 0.0039309133999978 0.8523575713999989 F F F
0.1432256068000015 0.2864512136999977 0.5733018912999981 F F F
0.5323249409999988 0.0646498820999994 0.6712332314000022 F F F
0.1404902625999966 0.8637678504000021 0.5714285713999985 F F F
0.5264974505999973 0.4702075758999982 0.6709513950000030 F F F
0.7232775877999984 0.8637678504000021 0.5714285713999985 F F F
0.9437101253000009 0.4702075758999982 0.6709513950000030 F F F
0.8575605759000027 0.7151211517000036 0.6272472695000033 F F F
0.1361761519000027 0.2723523037999982 0.6973712567000021 F F F
0.8602959201000004 0.1378045148999973 0.6291205894000029 F F F
0.1420036423999989 0.8667946099999995 0.6976530931000013 F F F
0.2775085947999969 0.1378045148999973 0.6291205894000029 F F F
0.7247909676000006 0.8667946099999995 0.6976530931000013 F F F
0.6662329541000034 0.3324659083000014 0.5790925471999984 F F F
0.3345532286000008 0.6691064570999998 0.6214566136000030 F F F
0.8109405171000006 0.6218810340999994 0.7413572186000010 F F F
0.2000398512999979 0.4000797026000029 0.8392885586999981 F F F
0.8082051728000010 0.1991976708999985 0.7394838987000014 F F F
0.1942123608000017 0.8056373964000016 0.8390067222999988 F F F
0.3909924980999975 0.1991976708999985 0.7394838987000014 F F F
0.6114250355999999 0.8056373964000016 0.8390067222999988 F F F
0.5252754861000000 0.0505509721999999 0.7953025967999992 F F F
0.8038910620999999 0.6077821241999999 0.8654265839999979 F F F
0.5280108302999977 0.4732343354000008 0.7971759166999988 F F F
0.8097185526000032 0.2022244305000029 0.8657084203999972 F F F
0.9452235051000031 0.4732343354000008 0.7971759166999988 F F F
0.3925058777999979 0.2022244305000029 0.8657084203999972 F F F
0.3339478644000025 0.6678957287999978 0.7471478745000013 F F F
0.0022681387999981 0.0045362776000033 0.7895119408999989 F F F
0.4424646103797005 0.8880514820435826 0.8972312923408515 T T T
0.1423939365121906 0.0062016431207598 0.0059411945494478 T T T
0.4821810262287459 0.5550685724699790 0.9193576819961410 T T T
0.7538357337399846 0.9616089447080686 0.9816918646616686 T T T
0.0457243864567506 0.5256250149911906 0.9128103519475359 T T T
0.1707133628736496 0.3923734715600378 0.9778915995691358 T T T
0.5772478537489215 0.0961426397958280 0.0463719093796970 T T T
0.3493596008389261 0.8404596585837956 0.9670314612159254 T T T
0.4792365412675212 0.5066752192433376 0.0424752838425586 T T T
0.8216632785323627 0.6214137818419263 0.9740717001740139 T T T
0.1718485604731591 0.6505593688317901 0.0428797250384129 T T T
0.0350600327916705 0.9781416152741803 0.9245760576253481 T T T
0.9986668927581934 0.2208643708431919 0.0552488811408108 T T T
```

no Mn + OH

```
  1.0000000000000000
    7.1831005684999996    0.0000000000000000    0.0000000000000000
   -3.5915502842999998    6.2436617694000001    0.0000000000000000
    0.0000000000000000    0.0000000000000000   35.0000000000000000
  Mn   Ru   Y    O    H
    4    8    8   41    1
Selective dynamics
Direct
0.3342505465000016 0.1685010929000015 0.6843022441000031 F F F
0.8342505465000016 0.1685010929000015 0.6843022441000031 F F F
0.0019654566999989 0.5039309133999978 0.8523575713999989 F F F
0.5019654566999989 0.5039309133999978 0.8523575713999989 F F F
0.9118753047083740 0.9038122899368835 0.9724561747962235 T T T
0.3164654117992378 0.8554553443353627 0.0172468521301871 T T T
0.8342505465000016 0.6685010929000015 0.6843022441000031 F F F
0.0003930912999976 0.0007861826999971 0.6002745803999971 F F F
0.5019654566999989 0.0039309133999978 0.8523575713999989 F F F
0.6681080015999967 0.3362160032000006 0.7683299077000001 F F F
0.1084081607036910 0.3394673139361220 0.0265846201418316 T T T
0.2953620322912996 0.5914880144902759 0.9348434297996556 T T T
0.0003930912999976 0.5007861826999971 0.6002745803999971 F F F
0.5003930912999976 0.5007861826999971 0.6002745803999971 F F F
0.5003930912999976 0.0007861826999971 0.6002745803999971 F F F
0.3342505465000016 0.6685010929000015 0.6843022441000031 F F F
0.6681080015999967 0.8362160032000006 0.7683299077000001 F F F
0.1681080015999967 0.8362160032000006 0.7683299077000001 F F F
0.1681080015999967 0.3362160032000006 0.7683299077000001 F F F
0.0019654566999989 0.0039309133999978 0.8523575713999989 F F F
0.1432256068000015 0.2864512136999977 0.5733018912999981 F F F
0.5323249409999988 0.0646498820999994 0.6712332314000022 F F F
0.1404902625999966 0.8637678504000021 0.5714285713999985 F F F
0.5264974505999973 0.4702075758999982 0.6709513950000030 F F F
0.7232775877999984 0.8637678504000021 0.5714285713999985 F F F
0.9437101253000009 0.4702075758999982 0.6709513950000030 F F F
0.8575605759000027 0.7151211517000036 0.6272472695000033 F F F
0.1361761519000027 0.2723523037999982 0.6973712567000021 F F F
0.8602959201000004 0.1378045148999973 0.6291205894000029 F F F
0.1420036423999989 0.8667946099999995 0.6976530931000013 F F F
0.2775085947999969 0.1378045148999973 0.6291205894000029 F F F
0.7247909676000006 0.8667946099999995 0.6976530931000013 F F F
0.6662329541000034 0.3324659083000014 0.5790925471999984 F F F
0.3345532286000008 0.6691064570999998 0.6214566136000030 F F F
0.8109405171000006 0.6218810340999994 0.7413572186000010 F F F
0.2000398512999979 0.4000797026000029 0.8392885586999981 F F F
0.8082051728000010 0.1991976708999985 0.7394838987000014 F F F
0.1942123608000017 0.8056373964000016 0.8390067222999988 F F F
0.3909924980999975 0.1991976708999985 0.7394838987000014 F F F
0.6114250355999999 0.8056373964000016 0.8390067222999988 F F F
0.5252754861000000 0.0505509721999999 0.7953025967999992 F F F
0.8038910620999999 0.6077821241999999 0.8654265839999979 F F F
0.5280108302999977 0.4732343354000008 0.7971759166999988 F F F
0.8097185526000032 0.2022244305000029 0.8657084203999972 F F F
0.9452235051000031 0.4732343354000008 0.7971759166999988 F F F
0.3925058777999979 0.2022244305000029 0.8657084203999972 F F F
0.3339478644000025 0.6678957287999978 0.7471478745000013 F F F
0.0022681387999981 0.0045362776000033 0.7895119408999989 F F F
0.4474875877960047 0.8911306120339644 0.8974371504363148 T T T
0.1663487323834288 0.0006774707523310 0.9998708563242796 T T T
0.4837501068251407 0.5390219178408959 0.9130624126864968 T T T
0.8655074558281957 0.0908495947276403 0.0027989995837306 T T T
0.0604548225533164 0.5496614816034308 0.9127905227091049 T T T
0.1842044727074172 0.3707227799324899 0.9698012386055618 T T T
0.4593212001306509 0.9469076872329431 0.0583691429559830 T T T
0.4229888843948189 0.8288609230565021 0.9678080531627252 T T T
0.3370100974409959 0.3771274287665216 0.0459299745488559 T T T
0.7404897744168573 0.6554410041620037 0.9892325619789538 T T T
0.0705042118830447 0.5810206214940803 0.0246336855424394 T T T
0.9639912766283792 0.9669339861679751 0.9243018331800975 T T T
0.9223114470358115 0.2727431481375033 0.0695028672952712 T T T
0.8392942498848202 0.1164580837753435 0.0728162102399349 T T T
```

no Mn + OOH

```
  1.0000000000000000
    7.1831005684999996    0.0000000000000000    0.0000000000000000
   -3.5915502842999998    6.2436617694000001    0.0000000000000000
    0.0000000000000000    0.0000000000000000   35.0000000000000000
  Mn   Ru   Y    O    H
    4    8    8   42    1
Selective dynamics
Direct
0.3342505465000016 0.1685010929000015 0.6843022441000031 F F F
0.8342505465000016 0.1685010929000015 0.6843022441000031 F F F
0.0019654566999989 0.5039309133999978 0.8523575713999989 F F F
0.5019654566999989 0.5039309133999978 0.8523575713999989 F F F
0.0331395829530898 0.0955401061081338 0.9679076486447664 T T T
0.2705696611990248 0.8532358277694035 0.0206185187562885 T T T
0.8342505465000016 0.6685010929000015 0.6843022441000031 F F F
0.0003930912999976 0.0007861826999971 0.6002745803999971 F F F
0.5019654566999989 0.0039309133999978 0.8523575713999989 F F F
0.6681080015999967 0.3362160032000006 0.7683299077000001 F F F
0.0051430386391618 0.5003514353884873 0.0922428964273633 T T T
0.2974700533185937 0.6029364621150880 0.9348430533739238 T T T
0.0003930912999976 0.5007861826999971 0.6002745803999971 F F F
0.5003930912999976 0.5007861826999971 0.6002745803999971 F F F
0.5003930912999976 0.0007861826999971 0.6002745803999971 F F F
0.3342505465000016 0.6685010929000015 0.6843022441000031 F F F
0.6681080015999967 0.8362160032000006 0.7683299077000001 F F F
0.1681080015999967 0.8362160032000006 0.7683299077000001 F F F
0.1681080015999967 0.3362160032000006 0.7683299077000001 F F F
0.0019654566999989 0.0039309133999978 0.8523575713999989 F F F
0.1432256068000015 0.2864512136999977 0.5733018912999981 F F F
0.5323249409999988 0.0646498820999994 0.6712332314000022 F F F
0.1404902625999966 0.8637678504000021 0.5714285713999985 F F F
0.5264974505999973 0.4702075758999982 0.6709513950000030 F F F
0.7232775877999984 0.8637678504000021 0.5714285713999985 F F F
0.9437101253000009 0.4702075758999982 0.6709513950000030 F F F
0.8575605759000027 0.7151211517000036 0.6272472695000033 F F F
0.1361761519000027 0.2723523037999982 0.6973712567000021 F F F
0.8602959201000004 0.1378045148999973 0.6291205894000029 F F F
0.1420036423999989 0.8667946099999995 0.6976530931000013 F F F
0.2775085947999969 0.1378045148999973 0.6291205894000029 F F F
0.7247909676000006 0.8667946099999995 0.6976530931000013 F F F
0.6662329541000034 0.3324659083000014 0.5790925471999984 F F F
0.3345532286000008 0.6691064570999998 0.6214566136000030 F F F
0.8109405171000006 0.6218810340999994 0.7413572186000010 F F F
0.2000398512999979 0.4000797026000029 0.8392885586999981 F F F
0.8082051728000010 0.1991976708999985 0.7394838987000014 F F F
0.1942123608000017 0.8056373964000016 0.8390067222999988 F F F
0.3909924980999975 0.1991976708999985 0.7394838987000014 F F F
0.6114250355999999 0.8056373964000016 0.8390067222999988 F F F
0.5252754861000000 0.0505509721999999 0.7953025967999992 F F F
0.8038910620999999 0.6077821241999999 0.8654265839999979 F F F
0.5280108302999977 0.4732343354000008 0.7971759166999988 F F F
0.8097185526000032 0.2022244305000029 0.8657084203999972 F F F
0.9452235051000031 0.4732343354000008 0.7971759166999988 F F F
0.3925058777999979 0.2022244305000029 0.8657084203999972 F F F
0.3339478644000025 0.6678957287999978 0.7471478745000013 F F F
0.0022681387999981 0.0045362776000033 0.7895119408999989 F F F
0.4409209299953534 0.8910401619565945 0.8981787451164437 T T T
0.1805720989344146 0.0563066740680130 0.0094342535434156 T T T
0.4964330353671598 0.5662881286053811 0.9129932731982655 T T T
0.8528985393513366 0.2295896158909394 0.0963430886346865 T T T
0.0497451053879866 0.5136607346846176 0.9112875182601566 T T T
0.2223129940414620 0.3926457498797868 0.9739329721201199 T T T
0.5118049283050259 0.9675487852755474 0.0436455294229025 T T T
0.3320646165040688 0.8222458489600197 0.9681989798897566 T T T
0.2761334321183093 0.5555782393189642 0.1103940003244194 T T T
0.7942840274551080 0.0522276216712395 0.9845594654654257 T T T
0.0683179481108201 0.5765560786248091 0.0392069607785416 T T T
0.0854132066306719 0.0288542052179864 0.9231859423259298 T T T
0.9499249182711360 0.6854600513518772 0.1109934716053072 T T T
0.4236956065721245 0.5533846496826004 0.0828810369465946 T T T
0.5475558804907635 0.6994607109566763 0.0855865822812045 T T T
```

S2. Supplementary References

1. Martínez-Coronado, R., Retuerto, M., Fernández, M. T. & Alonso, J. A. Evolution of the crystal and magnetic structure of the R 2MnRuO7 (R = Tb, Dy, Ho, Er, Tm, Yb, Lu, and Y) family of pyrochlore oxides. *Dalt. Trans.* **41**, 8575–8584 (2012).
2. McCrory, C. C. L. L., Jung, S., Peters, J. C. & Jaramillo, T. F. Benchmarking Heterogeneous Electrocatalysts for the Oxygen Evolution Reaction. *J. Am. Chem. Soc.* **135**, 16977–16987 (2013).
3. Yang, L. *et al.* Efficient oxygen evolution electrocatalysis in acid by a perovskite with face-sharing IrO6 octahedral dimers. *Nat. Commun.* **9**, 5236 (2018).
4. Lin, Y. *et al.* Chromium-ruthenium oxide solid solution electrocatalyst for highly efficient oxygen evolution reaction in acidic media. *Nat. Commun.* **10**, 162 (2019).
5. Suntivich, J., Gasteiger, H. A., Yabuuchi, N. & Shao-Horn, Y. Electrocatalytic Measurement Methodology of Oxide Catalysts Using a Thin-Film Rotating Disk Electrode. *J. Electrochem. Soc.* **157**, B1263 (2010).
6. Retuerto, M. *et al.* Structural effects of LaNiO3 as electrocatalyst for the oxygen reduction reaction. *Appl. Catal. B Environ.* **203**, 363–371 (2017).
7. Morgan, D. J. Resolving ruthenium: XPS studies of common ruthenium materials. *Surf. Interface Anal.* **47**, 1072–1079 (2015).
8. Mlynarczyk, M. *et al.* Surface layer of SrRuO3 epitaxial thin films under oxidizing and reducing conditions. *J. Appl. Phys.* **101**, 023701 (2007).
9. Lin, L., Starostin, S. A., Li, S., Khan, S. A. & Hessel, V. Synthesis of yttrium oxide nanoparticles via a facile microplasma-assisted process. *Chem. Eng. Sci.* **178**, 157–166 (2018).
10. Gougousi, T. & Chen, Z. Deposition of yttrium oxide thin films in supercritical carbon dioxide. *Thin Solid Films* **516**, 6197–6204 (2008).
11. Näslund, L.-Å., Ingason, Á. S., Holmin, S. & Rosen, J. Formation of RuO(OH)2 on RuO2-Based Electrodes for Hydrogen Production. *J. Phys. Chem. C* **118**, 15315–15323 (2014).
12. Petrović, S., Rakić, V., Jovanović, D. M. & Baričević, A. T. Oxidation of CO over Ru containing perovskite type oxides. *Appl. Catal. B Environ.* **66**, 249–257 (2006).
13. Gaur, S., Pakhare, D., Wu, H., Haynes, D. J. & Spivey, J. J. CO2 Reforming of

CH4 over Ru-Substituted Pyrochlore Catalysts: Effects of Temperature and Reactant Feed Ratio. *Energy and Fuels* **26**, 1989–1998 (2012).

14. Möckl, M. *et al.* Durability Testing of Low-Iridium PEM Water Electrolysis Membrane Electrode Assemblies. *J. Electrochem. Soc.* **169**, 064505 (2022).
15. Seitz, L. C. *et al.* A highly active and stable IrOx/SrIrO3 catalyst for the oxygen evolution reaction. *Science* **353**, 1011–1014 (2016).
16. Raman, A. S., Patel, R. & Vojvodic, A. Surface stability of perovskite oxides under OER operating conditions: A first principles approach. *Faraday Discuss.* **229**, 75–88 (2021).
17. Seh, Z. W. *et al.* Combining theory and experiment in electrocatalysis: Insights into materials design. *Science* **355**, eaad4998 (2017).
