# Supplementary material for: Active and durable R2MnRuO7 pyrochlores with low Ru content for acidic oxygen evolution
Source: Nat Commun. 2023 Apr 10;14:2010. doi: 10.1038/s41467-023-37665-9 (PMC10086044; doi:10.1038/s41467-023-37665-9)
Supplement: Supplementary file 2 — Peer Review File [file 41467_2023_37665_MOESM2_ESM.pdf]

nature portfolio

Peer Review File

Active and durable R2MnRuO7 pyrochlores with low Ru content for acidic oxygen evolution

Open Access This file is licensed under a Creative Commons Attribution 4.0 International License, which permits use, sharing, adaptation, distribution and reproduction in any medium or format, as long as you give appropriate credit to the original author(s) and the source, provide a link to the Creative Commons license, and indicate if changes were made. In the cases where the authors are anonymous, such as is the case for the reports of anonymous peer reviewers, author attribution should be to 'Anonymous Referee' followed by a clear attribution to the source work. The images or other third party material in this file are included in the article's Creative Commons license, unless indicated otherwise in a credit line to the material. If material is not included in the article's Creative Commons license and your intended use is not permitted by statutory regulation or exceeds the permitted use, you will need to obtain permission directly from the copyright holder. To view a copy of this license, visit http://creativecommons.org/licenses/by/4.0/.

REVIEWER COMMENTS</B>

Reviewer #1 (Remarks to the Author):

Recommendation: Suitable for publication after major revisions.
The authors studied some R2MnRuO7 based catalysts, with R= Y, Tb and Dy, for OER in acidic media. They demonstrated how Y2MnRuO7 is the most stable catalyst among the ones studied, maintaining a potential of 1.5 V at 10 mA cm-2 for 40 h in half-cell experiments. They also tested the catalyst in a full device (PEMWE) for 4h applying a constant current density of 1A cm-2.
Suggesting of the reviewer:
• Move table S3 into main text, as it contains references that are listed in the manuscript and not in the supporting informations;
• In Table S3, the authors compare their results (in terms of current density J / mA cm-2) with other works reported in literature (for example ref. [19]): the data referred to the work [19] seems to be reported in a wrong way, it could be due to the fact that current density was not expressed versus geometric area but versus BET area of the samples. Please, this fact should be verified from supporting information of work [19] (Figure S9 vs. Figure S10).
• A similar problem is present in Table S3 under “Ru mass specific activity” (A g-1Ru): where did the authors find the data of work [19]? From Figure S10c of that work? What does the “*” refer to?
• Y2MnRuO7 was tested in a PEMWE: the present work is focused on the major stability of the Y2MnRuO7 based catalyst, so the authors should demonstrate this aspect also in the full device registering longer stability test, and comparing this measure with a benchmark catalyst (IrO2 at similar loading of Ru) at the same cell conditions. See for example: Maximilian Möckl et al 2022 J. Electrochem. Soc. 169 064505. 10.1149/1945-7111/ac6d14

Reviewer #2 (Remarks to the Author):

The manuscript by Galyamin et al. describes RuMn pyrochlores as the active catalysts for the oxygen evolution reaction with reduced noble metal content. The work is generally carefully done, however, in referee’s opinion, it lacks fundamental insights and novelty required to meet the standards of a premier journal. Indeed, mixed ruthenium-manganese oxides were recently reported to be active in acidic OER [Energy Environ. Sci., 2022, 15, 2356]; specific OER activity does not look superior to the similar published materials, whereas mass activity is significantly lower than that of the carbon-supported single atom site catalysts [e.g. Nat. Commun., 2019, 4849]. Moreover, manuscript does not report conclusions which could be applied to other system or guide the materials design. However, if the authors can clarify the above concerns and address the specific comments below, Referee can revise his opinion.

1) P.3.: “there is only one OER catalyst in which Ru was partially replaced with a non-noble metal

cation…" This statement is not correct: there are many works on the metal-substituted RuO_2 rutile oxide [e.g. Chem. Mater., 2020, 32, 6150; Angew. Chem. Int. Ed., 2010, 49, 4813, etc.]. There are also studies on the low-content Ru oxides where Ru sites are installed into the structure of the non-noble metal-based oxide [e.g. Nat. Commun., 2021, 4587] (such systems however are typically studied in alkaline conditions).

2) Activity of R_2MnRuO_7 catalysts is compared to $Y_2Ru_2O_7$, selected by authors as a benchmark, however it was shown that some ruthenium pyrochlores with other A sites (e.g. $Cu_xY_{2-x}Ru_2O_7$) are more active than $Y_2Ru_2O_7$ [JACS, 2020, 142, 7883]. This has to be mentioned in the text and comparison of the OER performance has to be expanded to include those more active compositions.
Study would also benefit if the comparison of the specific activities (oxide surface area-normalized) between R_2MnRuO_7 and literature references (where available) is provided in the manuscript in the form of Tafel plots.

3) It should be discussed in the text why the activity of R_2MnRuO_7 depends on R if the active site is the same $RuMnO_x$ layer for all compositions.

4) ICP analysis. Authors only provide the concentration of the dissolved elements in the electrolyte. What fraction of the total loading does the dissolved amount correspond to? What is the minimum concentration that can be detected by the instrument with respect to the total loading? It would help to estimate the thickness of the amorphous/reconstructed surface layer.

Minor comments:
- Fig. 6: where the experimental data for delta(G) – delta(GOH) for the YMRO(exp) data point are coming from?
- Acronym ESSI (P. 19) should be explained.

Reviewer #3 (Remarks to the Author):

The manuscript describes a detailed study of the structure-activity relationships of low Ru-containing lanthanide-RuMn-pyrochlore electrocatalyst for electrochemical oxygen evolution reaction (OER) in acidic media. It is demonstrated that $R_2MnRu_2O_7$ exhibits higher OER activity than $R_2Ru_2O_7$ in acidic media (R=lanthanide elements). The work has been carried out comprehensively and skillfully. This work has added substantial knowledge and information about the performance of pyrochlores with regards to OER in acidic media. I recommend this manuscript for publication with minor modifications.
Comment 1: The change of ECSA with the OER cycle has not been reported. It will be helpful to readers if the authors provide the details of how ECSA is changing with OER cycles.
Comment 2: There is additional reported literature regarding OER activity of rare-earth Ir and Ru-pyrochlores (see Nano Lett. 2017, 17, 3974-3981, Acc. Chem. Res. 2022, 55, 16, 2191–2200) in acidic media. Previously, OER performances with mixed Ru-pyrochlores with substitution of Fe, Ni and Cu have

been recorded. Please compare their OER activity with R2MnRuO7.
Comment 3. A stability test of PEMWE for only 4 hours is not good enough to evaluate its viability. At least, a minimum stability test of 12 hours is useful to assess its stability.

RESPONSE TO REVIEWERS' COMMENTS

<u>Reviewer 1</u>

Recommendation: Suitable for publication after major revisions.

The authors studied some R_2MnRuO_7 based catalysts, with R = Y, Tb and Dy, for OER in acidic media. They demonstrated how Y_2MnRuO_7 is the most stable catalyst among the ones studied, maintaining a potential of 1.5 V at 10 mA cm^{-2} for 40 h in half-cell experiments. They also tested the catalyst in a full device (PEMWE) for 4 h applying a constant current density of 1 A cm^{-2}.

Suggesting of the reviewer:

Question 1.

Move Table S3 into main text, as it contains references that are listed in the manuscript and not in the supporting information.

Response 1.

We thank Reviewer 1 for this observation. After the reviewer's comment, we have moved Table S3 into the main text as Table 1. After this change, the rest of the tables have been renumbered in the revised manuscript.

Question 2.

In Table S3, the authors compare their results (in terms of current density J / mA cm^{-2}) with other works reported in literature (for example ref. [19]): the data referred to the work [19] seems to be reported in a wrong way, it could be due to the fact that current density was not expressed versus geometric area but versus BET area of the samples. Please, this fact should be verified from supporting information of work [19] (Figure S9 vs. Figure S10).

Response 2.

Based on Reviewer 1's comment, we have double-checked the current density values provided in the original Table S3 (Table 1 in the revised manuscript). Unless otherwise stated, the values in Table 1 are reported as mA cm^{-2}_{geo}. For instance, the values in Table 1 from reference 19 were taken from its Figure S10, not from Figure S9. If we understand correctly, the activity curves shown in Figure S10 from reference 19 are reported in mA cm^{2}_{geo}.

Question 3.

A similar problem is present in Table S3 under "Ru mass specific activity" (A g^{-1} Ru): where did the authors find the data of work [19]? From Figure S10c of that work? What does the "*" refer to?

Response 3.

Firstly, we apologize for not having indicated the meaning of "*" in the original manuscript. The symbol "*" placed beside some references indicates that the data reported in our manuscript were extracted by digitalization of the figures reported therein. This is now indicated in Table 1 in the revised manuscript (Former Table S3). The Ru

mass specific activity from reference 19 was extracted from its Figure S10d. We used data from this figure because it reports activity data after 10 cycles, when the reconstruction of the surface occurs. We note that the activity data for our catalysts were reported after 100 OER cycles. Page 10 in the manuscript now reads: "*We note that Table 1 contains the highest OER activity value reported for each catalyst in the cited manuscripts, regardless of the cycle at which such activity was recorded.*" The cycle at which the activity is reported is now indicated in Table 1 by the superscritps † and ‡.

Question 4.

Y_2MnRuO_7 was tested in a PEMWE: the present work is focused on the major stability of the Y_2MnRuO_7 based catalyst, so the authors should demonstrate this aspect also in the full device registering longer stability test, and comparing this measure with a benchmark catalyst (IrO2 at similar loading of Ru) at the same cell conditions. See for example: Maximilian Möckl et al 2022 J. Electrochem. Soc. 169 064505. 10.1149/1945-7111/ac6d14

Response 4.

Reviewer 1 raises an interesting point here. We are aware that the durability of Y_2MnRuO_7 in PEMWE configuration would be lower than with state-of-t-he-art (SoA) Ir-based catalysts. However, Y_2MnRuO_7 displays both high activity and durability in the context of Ru-based (Ir-free) catalysts. Durability is the most critical issue of Ru-based catalysts for the OER. In fact, they are so unstable that most of Ru-based catalysts cannot even be properly tested in PEMWEs. This is true even for highly loaded Ru catalyst coated membranes (CCMs). The few catalysts that have been tested in PEMWEs are measured with high Ru loadings (2 $mg_{Ru}cm^{-2}$ or higher) and low currents, typically 500 $mAcm^{-2}$ or lower. However, since Y_2MnRuO_7 displays high activity and durability in RDE, it allowed us to assemble a CCM with a remarkably low Ru loading of 0.2 $mg_{Ru}cm^{-2}$, a value that compares well with those of SoA Ir-based catalysts, and at a high current up to 1 A cm^{-2}. As said in our manuscript, under such severe conditions, the catalyst is not stable for long-time operation, showing a stable performance for only a few hours. Nonetheless, in the context of Ru-based catalysts, this result is a step forward in the design and test of Ru-based catalysts as anodes for PEMWEs.

Following Reviewer 1's comment, we now provide in Figure 7b longer durability tests of an MEA with Y_2MnRuO_7 (0.2 mg_{Ru}/cm^2) at 0.2, 0.5 and 1.0 A cm^{-2}. We performed the 0.2 A cm^{-2} stability test to compare it with a similar test reported in *Nature Materials, 2023, 22, 100-108* (Reference 10 in the main manuscript) for assessing the stability of Ru-based catalysts, but in our case with a 10 times lower Ru loading.

After Reviewer 1's suggestion, we also tested a CCM with an Ir anode under similar reaction conditions, 0.2 mg_{Ir} cm^{-2} at 0.2 and 0.5 Acm^{-2}. As observed in the figure below (Figure R1 here, Figure S6 in the revised manuscript), Ir_{black} and Y_2MnRuO_7 show similar durability at low current (0.2 A cm^{-2}). The MEA with the Ir catalyst is more stable at 0.5 A cm^{-2}, but shows higher potential values than the Y_2MnRuO_7 catalyst during the first 8 h of the experiment. This comparison has been included in the Supporting Information as "*S.1.9. Comparison of a PEMWE with an Y_2MnRuO_7 anode and a PEMWE with an Ir_{black} anode.*"

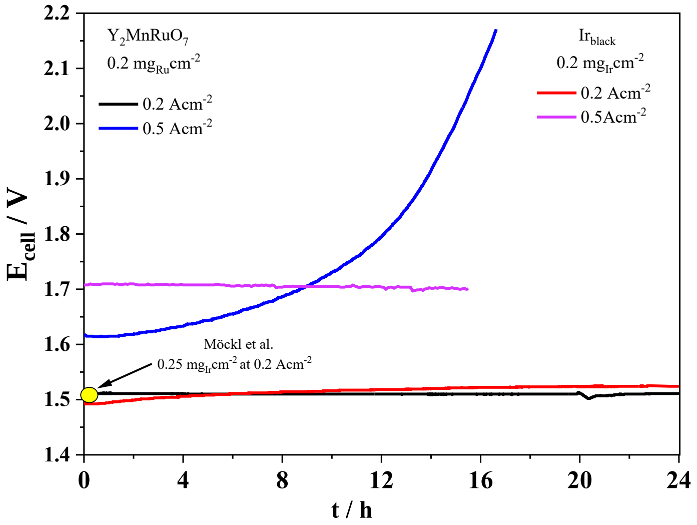

Figure R1. Stability tests of PEMWE using Y_2MnRuO_7 and Ir_{black} as anodes at constant current densities of 0.2 and 0.5 A cm^{-2} at 80 °C and 1 bar.

Reviewer 2

Question 1.

The manuscript by Galyamin et al. describes RuMn pyrochlores as the active catalysts for the oxygen evolution reaction with reduced noble metal content. The work is generally carefully done, however, in referee's opinion, it lacks fundamental insights and novelty required to meet the standards of a premier journal. Indeed, mixed ruthenium-manganese oxides were recently reported to be active in acidic OER [Energy Environ. Sci., 2022, 15, 2356]; specific OER activity does not look superior to the similar published materials, whereas mass activity is significantly lower than that of the carbon-supported single atom site catalysts [e.g. *Nat. Commun., 2019, 4849*].

Response 1.

Reviewer 2 is right and we are aware that the Ru-Mn oxide reported in *Energy Environ. Sci., 2022, 15,* displays high OER activity in acidic electrolyte. It is also true that the Ru-N-C catalyst reported in *Nat. Commun., 2019, 4849* is a salient catalyst with higher Ru mass specific activity than our catalyst measured in 0.5 M H_2SO_4. However, the structure of the catatlys reported in the latter reference (single atom Ru in a N-C matrix) is completely different to that of the catalysts reported in our work, namely Ru-mixed oxides. Besides, none of the papers mentioned by Reviewer 2 studies the catalytic performance in MEAs. However, based on Reviewer 2's comment, and in view of their high activity in acidic electrolytes, we have cited both papers in the revised manuscript, as references 11 and 12.

In addition, whereas comparable OER activities in RDE are reported, our work goes further and reports catalyst activity in an actual PEMWE with much lower Ru loading (0.2 mg_{Ru}/cm^2) than previously reported Ru-based electrodes.

Question 2.

Moreover, manuscript does not report conclusions which could be applied to other system or guide the materials design. However, if the authors can clarify the above concerns and address the specific comments below, Referee can revise his opinion.

Response 2.

We thank Reviewer 2 for letting us know that we were not clear enough in our conclusions. We have improved the discussion to better explain how we found significant results to guide the community in the search for novel approaches to improve Ru catalysts.

First of all, our work offers a comprehensive computational study on the origin of the high activity of Ru pyrochlore catalysts. We conclude that partial Y dissolution is necessary for a high OER activity. Besides, Mn is shown to be instrumental for the high electrocatalytic activity observed in experiments, as Mn-Ru-O ensembles at the surface of the catalysts are responsible for the high OER performance. In fact, the OER activity is shown to decrease alongside the Mn content at the surface and we rationalize the effect on the basis of the electrocatalytic symmetry of the OER pathway on Ru sites. We are convinced that a 1:1 local ratio of Ru:Mn in a pyrochlore network in which the large cation has been partially dissolved, is a simple and descriptive guideline for the experimental and computational design of active OER catalysts.

Second, our experiments show the effect of having surface Ru-Mn-O ensembles: there is a significant decrease of Ru loading from the pyrochlore catalysts without compromising the activity and actually enhancing the durability. In fact, the performance of our material surpasses those of numerous other Ru-O surfaces.

Finally, we took our material with low Ru content a step beyond the conventional laboratory tests and showed that it works in a PEMWE with a loading of only 0.2 mg_{Ru} cm^{-2} at 0.2 A cm^{-2} during at least 25 h; at 0.5 A cm^{-2} during ca. 10 h and and at 1 A cm^{-2} during 4 h. We anticipate that our design guideline may inspire future works focused on (i) strategies to further stabilize surface Ru-Mn-O ensembles, and (ii) the testing of metals other than Mn.

We have changed the abstract in order to be clearer and more concise: "*These results encourage further investigation on Ru catalysts in which a partial replacement of Ru by inexpensive cations can enhance the OER performance*."

On page 21 we included the paragraph: "*In sum, our computational and experimental results indicate that Y_2MnRuO_7 pyrochlore is an excellent starting point to form an active $RuMnO_x$ surface. Besides, we show here that a thoughtful design of the pyrochlore phases with suitable (Ru,Mn)–O bonds and Ru/Mn-O-Ru/Mn angles can provide active pyrochlores with lower Ru content than $R_2Ru_2O_7$.*"

We have changed the conclusions to: "*This finding opens a route to further enhance the activity and durability of Ru mixed oxides by partly replacing Ru by other cations, as potential electrocatalysts for the anode of PEMWEs with lower content of noble metals*."

Question 3.

"there is only one OER catalyst in which Ru was partially replaced with a non-noble metal cation…" This statement is not correct: there are many works on the metal-substituted RuO2 rutile oxide [e.g. Chem. Mater., 2020, 32, 6150; Angew. Chem. Int. Ed., 2010, 49, 4813, etc.]. There are also studies on the low-content Ru oxides where Ru sites are installed into the structure of the non-noble metal-based oxide [e.g. Nat. Commun., 2021, 4587] (such systems however are typically studied in alkaline conditions).

Response 3.

We thank Reviewer 2 for bringing this point to our attention. The original sentence was not correct. We meant decreasing Ru in pyrochlores and not in oxides in general.

Therefore, in the introduction we have included some references about the general improvement of activity in Ru oxides when Ru is replaced by non noble metals: "*The full replacement of Ir by other metals is more challenging. Ru nanoparticles and $Ru_{1-y}M_yO_x$ based compounds;[10-12]and mixed Ru oxides….*"

We have also modified the text on page 3, which now reads: "*To the best of our knowledge, there is one OER catalyst in which Ru is partially replaced by non noble metal cations in the pyrochlore structure, $Y_2[Ru_{1.6}Y_{0.4}]O_{7-\delta}$, showing higher activity than the parent $Y_2Ru_2O_{7-\delta}$ but without stability studies and a detailed understanding of the origin of its performance.[30]*"

It is also true that a number of Ru-based electrocatalysts have been reported to be active for the OER in alkaline media. However, in such media the reduction of noble materials is far from being a critical issue, since many catalysts without critical materials are excellent OER catalysts. In fact, some state-of-the-art catalysts in alkaline media are based on Ni and Fe).

Question 4.

Activity of R_2MnRuO_7 catalysts is compared to $Y_2Ru_2O_7$, selected by authors as a benchmark, however it was shown that some ruthenium pyrochlores with other A sites (e.g. $Cu_xY_{2-x}Ru_2O_7$) are more active than $Y_2Ru_2O_7$ [JACS, 2020, 142, 7883]. This has to be mentioned in the text and comparison of the OER performance has to be expanded to include those more active compositions.

Response 4.

We thank Reviewer 2 for bringing this reference to our attention. Pyrochlores as electrocatalysts for oxygen evolution are a trending topic in the literature and a number of papers focusing on improving their activity and durability have been published in the past few years. Following Reviewer 2's suggestion, we have modified our manuscript to include the reference dealing with $Cu_xY_{2-x}Ru_2O_7$ catalysts in the introduction section and in Table 1, as reference 29.

Question 5.

Study would also benefit if the comparison of the specific activities (oxide surface area-normalized) between R_2MnRuO_7 and literature references (where available) is provided in the manuscript in the form of Tafel plots.

Response 5.

In Table 1 we report the Tafel plots of our catalysts and of various catalysts in the literature. It is worth noting that the Tafel slopes should not depend on whether the activities are reported in terms of geometric areas or oxide surface areas, since the slope would be the same. Note also that in Figure S2c-d we show the specific activities (normalized to A_s and ECSA) of the three catalysts reported in our work.

Question 6.

It should be discussed in the text why the activity of R_2MnRuO_7 depends on R if the active site is the same RuMnOx layer for all compositions.

Response 6.

Our theoretical study reveals that the OER activity is related to the Ru-Mn-O surface. However, we note that the actual structure/morphology of the surface formed after reconstruction may be different for each catalyst, i.e. for each R cation. For instance, Ru-Mn-O surface ensembles with different Ru/Mn-O distances and Ru/Mn-O-Ru/Mn angles can be formed depending on the different R atoms in the A site of the pyrochlore.

This is not unique to the catalysts reported in this work, and a similar feature can be found in the literature. For instance, Ir catalysts based upon mixed iridium oxides display different OER activity, yet after reconstruction they tend to from similar IrO_xOH_y phases. See, for instance, reference *Nat. Commun. 2022, 13:7935.*

As for the particular role of Y, we would like to bring Reviewer 2's attention to Figure 2b. Note how all of the most active pyrochlores have Y in the A site of the structure, so it is clear that Y has the ideal size and charge to accommodate the most active Ru sites among the pyrochlores.

Trying to give additional insight about the different activities found for the different R cations we slightly changed the text:

- On page 12 we improved the explanation to: "*The activities reached by the catalysts can be related to their crystallographic structure, including (Ru,Mn)–O distances, angles, oxygen vacancies, etc. For instance,* Y_2MnRuO_7 *has slightly shorter (Ru,Mn)–O bonds and slightly less bent Ru/Mn-O-Ru/Mn angles (see Table S2) than* Dy_2MnRuO_7 *and* Tb_2MnRuO_7*……*
- On page 13: "*Note that the OER activities of* $R_2Ru_2O_7$ *pyrochlores with R = Y are higher than with other cations (see Figure 2b).*"
- On page 21 we have included: "*In sum, our computational and experimental results indicate that* Y_2MnRuO_7 *pyrochlore is an excellent starting point to form an active* $RuMnO_x$ *surface. Besides, in this work we show that a thoughtful design of the pyrochlore phases with suitable (Ru,Mn)–O bonds and Ru/Mn-O-Ru/Mn angles can provide active pyrochlores with lower Ru content than* $R_2Ru_2O_7$."

Question 7.

ICP analysis. Authors only provide the concentration of the dissolved elements in the electrolyte. What fraction of the total loading does the dissolved amount correspond to? What is the minimum concentration that can be detected by the instrument with respect to the total loading? It would help to estimate the thickness of the amorphous/reconstructed surface layer.

Response 7.

Reviewer 2 is correct and we should have provided more information about the ICP results. For that reason, we have conducted additional ICP-OES experiments in the electrolyte.

- We have now included a section in the Supporting Information "*S.1.8. Inductively Coupled Plasma Optical Emission Spectrometry (ICP-OES)*" with the information we got from the ICP experiments. We provided the maximum concentrations that could be dissolved (considering the catalyst loading and the electrolyte volume) and gave the weight percentages dissolved of each cation.
- On page 19 we modified the discussion to: "*Finally, we determined the concentration of the cations in the electrolyte after 100 and 6000 OER cycles with* Y_2MnRuO_7 *(Table S5). After 100 cycles, we recorded a small dissolution of Y (8 wt.%) and Mn (4 wt.%). The amount of dissolved Ru is below the detection limit. After 6000 cycles, when the OER activity commences to decline, the dissolution of cations is more severe, and the fraction of dissolved metal amounts to 43, 18 and 9 wt.% for Y, Mn and Ru, respectively. These results confirm the stability of the pyrochlore during the first 4000-5000 reaction cycles. They also confirm the faster dissolution of Y during the early reaction cycles, which is in line with the formation of Ru-Mn-O surface ensembles.*"

Minor comments:

Question 8.

Fig. 6: where the experimental data for delta(G) – delta(GOH) for the YMRO(exp) data point are coming from?

Response 8.

As now explained in the caption of Figure 6, the experimental data in Figure S2c is used to calculate the corresponding value of $\Delta G_O - \Delta G_{OH}$ by means of the method in references [16,46]. The method uses the experimental OER overpotential corresponding to a current density of 1 mA cm^{-2}_{As}, which for YMRO is around 0.29 V. That overpotential is used to approximate the sought binding energy difference by means of the semiempirical volcano plot in [46]. In particular, 0.29 V corresponds to $\Delta G_O - \Delta G_{OH} \approx 1.53\ eV$, knowing that YMRO is on the strong-binding side of the volcano, as revealed by our DFT calculations.

Question 9.

Acronym ESSI (P. 19) should be explained.

Response 9.

ESSI stands for electrochemical-step symmetry index. The acronym is spelled out in the caption of Figure 6 and toward the end of section 2.8 (Computational details), where the formula used for its assessment is also provided. For clarity, in the revised manuscript we have also spelled out ESSI on p. 20, as requested by Reviewer 2. We have also added an explanatory arrow to the inset of Figure 6 to show how the OER activity increases as a function of ESSI (see Figure R2 below).

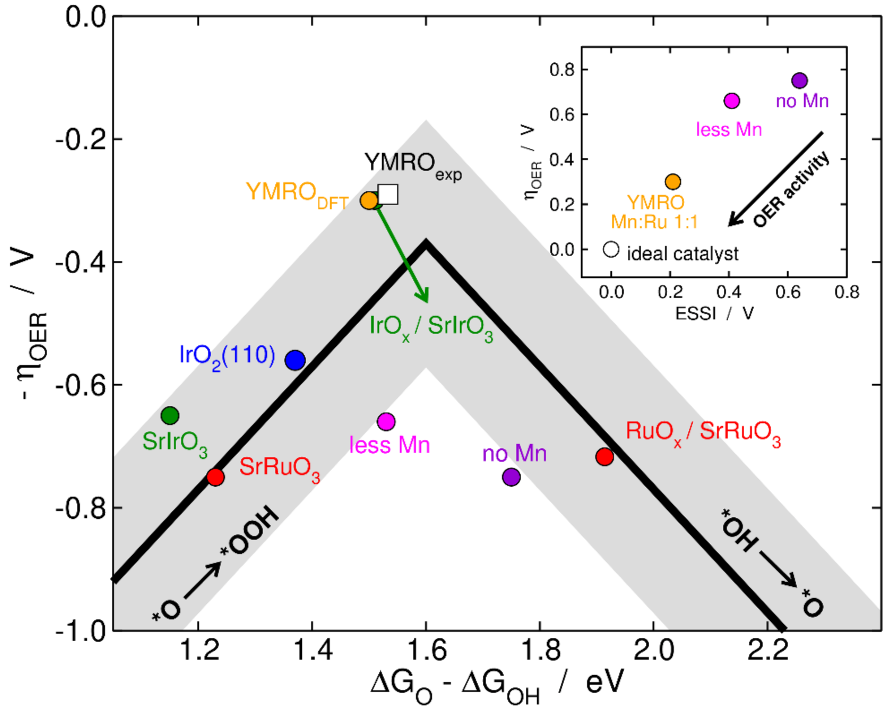

Figure R2. Volcano-type activity plot for the OER in acid for a variety of oxides, including IrO_2(110), pristine $SrIrO_3$ and $IrO_x/SrIrO_3$,[62] pristine $SrRuO_3$ and $RuO_x/SrRuO_3$,[16] and Y_2MnRuO_7 with a 1:1 ratio of Mn:Ru (denoted as YMRO), less Mn at the surface, and no Mn at the surface, see Figures S7-S9. The white

square ($YMRO_{exp}$) is the experimental data from Figure S2c adapted using a method reported elsewhere [16,46], see section S2. Inset: OER overpotential as a function of the electrochemical-step symmetry index (ESSI) for different Ru-containing catalysts. The ideal catalyst, which has ΔG_i = 1.23 eV and η_{OER} = 0 V, is provided for comparison.

Reviewer 3

The manuscript describes a detailed study of the structure-activity relationships of low Ru-containing lanthanide-RuMn-pyrochlore electrocatalyst for electrochemical oxygen evolution reaction (OER) in acidic media. It is demonstrated that $R_2MnRu_2O_7$ exhibits higher OER activity than $R_2Ru_2O_7$ in acidic media (R = lanthanide elements). The work has been carried out comprehensively and skillfully. This work has added substantial knowledge and information about the performance of pyrochlores with regards to OER in acidic media. I recommend this manuscript for publication with minor modifications.

Question 1.

The change of ECSA with the OER cycle has not been reported. It will be helpful to readers if the authors provide the details of how ECSA is changing with OER cycles

Response 1.

Motivated by Reviewer 3's comment we have conducted further experiments to determine the evolution of the ECSA with cycling. Thus, after a number of cycles, we determined the double layer capacitance (C_{dl}) by impedance spectroscopy, and the ECSA value. As shown in Figure S4 in the novel version of the manuscript, three regions with three different C_{dl} (and ECSA) can be observed. C_{dl} increases during the first OER cycles (ca. 500) with an average ECSA of ca. 9 m^2. This is the "activation region". Once stable, a constant C_{dl} value with ECSA of ca. 12 m^2 is observed during 5000 cycles. After 5000 cycles, both C_{dl} with ECSA of 3 m^2 and OER activity decline.

- All these results have been included as a novel section in the Supporting Information: "*S1.5. Assessment of the evolution of C_{dl} and ECSA with OER cycling*"

- Page 15, section 3.4 reads: "*First, we analysed the evolution of the electrochemical surface area (ECSA) during cycling. The ECSA is directly related to the double layer capacitances, C_{dl}, and can be determined by CV or EIS. As shown in Figure S3, the double layer capacitance increases during the first ca. 500 cycles, a feature associated with the reconstruction of the catalyst surface (see below). ECSA remains stable until ca. 5000 cycles, which is the region of stable OER activity, and finally drops with further cycling, coinciding with catalyst deactivation profile.*"

Question 2.

Comment 2: There is additional reported literature regarding OER activity of rare-earth Ir and Ru-pyrochlores (see Nano Lett. 2017, 17, 3974-3981, Acc. Chem. Res. 2022, 55, 16, 2191–2200) in acidic media. Previously, OER performances with mixed Ru-pyrochlores with substitution of Fe, Ni and Cu have been recorded. Please compare their OER activity with R_2MnRuO_7.

Reponse 2.

We thank Reviewer 3 for this comment. We have included the references that report activity in acidic media in the revised manuscript, and the activities are compared with those of R_2MnRuO_7 in the text and in Table 1. However, we did not include the Ru-based electrocatalysts reporting OER in alkaline media. This is simply because our current focus

is on acidic conditions and because highly active catalysts based on non noble metals exist for alkaline conditions.

Question 3.

A stability test of PEMWE for only 4 hours is not good enough to evaluate its viability. At least, a minimum stability test of 12 hours is useful to assess its stability.

Response 3.

We agree with this coment. In order to address Reviewer 3's request, the text has been mofified, and additional durability tests were performed. We note that in the literature Ru catalyst CCMs are rarely tested at current densities higther than 0.5 A cm^{-2} and always with loadings larger than 2 mg_{Ru} cm^{-2}. Our CCM was made with only 0.2 mg_{Ru} cm^{-2} in the anode and the polarization curve was recorded up to 2.0 A cm^{-2}, showing a non iR-corrected E_{cell} of ca 2.08 V. See Figure R3a (Novel Figure 7a in the revised manuscript).

We performed durability tests at 0.2, 0.5, and 1.0 A cm^{-2}. As observed in Figure R3b, we obtained a low degradation rate of 40 µA h^{-1} at 0.2 A cm^{-2} for at least 24 h. The stability of Ru-based catalysts in PEMWE at such low current density has been reported for other Ru-based catalysts although with ca. 10 times higher Ru loading, see *Nature Materials, 2023, 22, 100-108* (reference 10). Conversely, we tested our CCM at higher currents, namely 0.5 and 1.0 A cm^{-2}. After few hours, both experiments do not present important performance losses and the degradation rates are 7.5 mV h^{-1} and 22 mV h^{-1}, respectively, after which the degradation increases (see Figure S6). Those results were included in Figure R3b and included in the revised manuscript as Figure 7b.

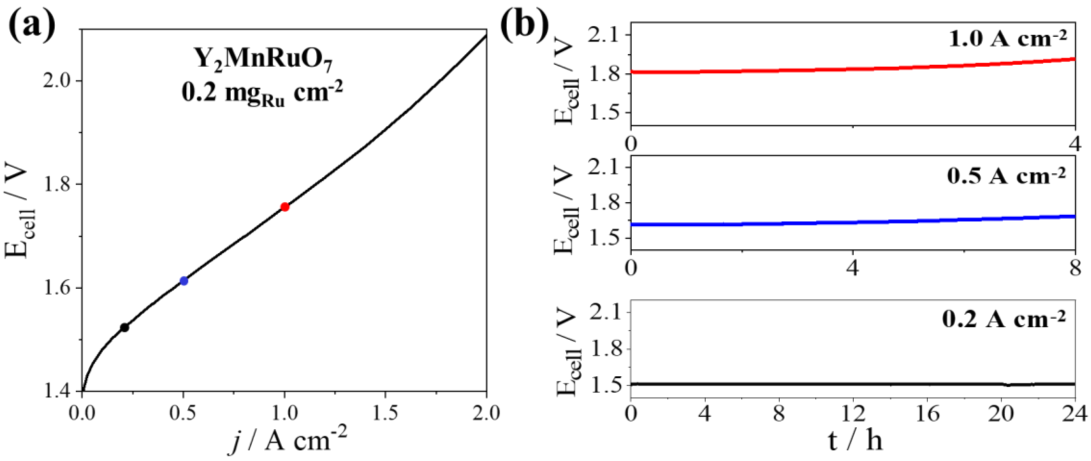

Figure R3. PEMWE measurements with Y_2MnRuO_7 anodes. (a) Cell potential (E_{cell}) as a function of the current density (j) recorded galvanostatically up to 2 A cm^{-2} at 80 °C and 1 bar. (b) Durability test of PEMWE at constant current density of 0.2, 0.5 and 1 A cm^{-2} at 80 °C and 1 bar.

REVIEWERS' COMMENTS

Reviewer #1 (Remarks to the Author):

Thanks to the authors for having answered the questions in a precise way; I consider the final work profoundly improved and suitable for publication. The additional durability measurements encourage further investigation on low loading Ru pyrochlores based catalysts for acidic OER.

Reviewer #2 (Remarks to the Author):

The authors expanded the manuscript and revised the discussion addressing Referee's comments. The manuscript can now be recommended for publication.

Reviewer #3 (Remarks to the Author):

The manuscript describes a detailed study of the structure-activity relationships of low Ru-containing lanthanide-RuMn-pyrochlore electrocatalyst for electrochemical oxygen evolution reaction (OER) in acidic media. It is demonstrated that R2MnRu2O7 exhibits higher OER activity than R2Ru2O7 in acidic media (R=lanthanide elements). The work has added substantial knowledge and information about the performance of pyrochlores with regards to OER in acidic media.The conclusions are supported by experimental and simulation results. The authors have addressed satisfactorily my queries.

POINT BY POINT RESPONSE TO REVIEWERS COMMENTS

Reviewer #1 (Remarks to the Author): Thanks to the authors for having answered the questions in a precise way; I consider the final work profoundly improved and suitable for publication. The additional durability measurements encourage further investigation on low loading Ru pyrochlores based catalysts for acidic OER.

Response reviewer 1. We thank reviewer 1 for his/her comments and for acknowledging our work.

Reviewer #2 (Remarks to the Author):vThe authors expanded the manuscript and revised the discussion addressing Referee's comments. The manuscript can now be recommended for publication.

Response to reviewer 2. We thank reviewer for his/her positive comments.

Reviewer #3 (Remarks to the Author):
The manuscript describes a detailed study of the structure-activity relationships of low Ru-containing lanthanide-RuMn-pyrochlore electrocatalyst for electrochemical oxygen evolution reaction (OER) in acidic media. It is demonstrated that R2MnRu2O7 exhibits higher OER activity than R2Ru2O7 in acidic media (R=lanthanide elements). The work has added substantial knowledge and information about the performance of pyrochlores with regards to OER in acidic media.The conclusions are supported by experimental and simulation results. The authors have addressed satisfactorily my queries.

Response to reviewer 3. We would like to thank the reviewer for his/her comments.
